# Cholesterol modulates type I/II TGF-β receptor complexes and alters the balance between Smad and Akt signaling in hepatocytes

**DOI:** 10.1038/s42003-023-05654-9

**Published:** 2024-01-02

**Authors:** Roohi Chaudhary, Laureen S. Goodman, Sai Wang, Anastasia Asimakopoulos, Ralf Weiskirchen, Steven Dooley, Marcelo Ehrlich, Yoav I. Henis

**Affiliations:** 1 Shmunis School of Biomedicine and Cancer Research, George S. Wise Faculty of Life Sciences, Tel Aviv University, 6997801 Tel Aviv, Israel; 2 Department of Neurobiology, George S. Wise Faculty of Life Sciences, Tel Aviv University, 6997801 Tel Aviv, Israel; 3 Department of Medicine II, University Medical Center Mannheim, Medical Faculty Mannheim, Heidelberg University, D-68167 Mannheim, Germany; 4 Institute of Molecular Pathobiochemistry, Experimental Gene Therapy and Clinical Chemistry (IFMPEGKC), RWTH Aachen University Hospital, D-52074 Aachen, Germany

**Keywords:** Growth factor signalling, Membrane structure and assembly

## Abstract

Cholesterol mediates membrane compartmentalization, affecting signaling *via* differential distribution of receptors and signaling mediators. While excessive cholesterol and aberrant transforming growth factor-β (TGF-β) signaling characterize multiple liver diseases, their linkage to canonical *vs*. non-canonical TGF-β signaling remained unclear. Here, we subjected murine hepatocytes to cholesterol depletion (CD) or enrichment (CE), followed by biophysical studies on TGF-β receptor heterocomplex formation, and output to Smad2/3 *vs*. Akt pathways. Prior to ligand addition, raft-dependent preformed heteromeric receptor complexes were observed. Smad2/3 phosphorylation persisted following CD or CE. CD enhanced phospho-Akt (pAkt) formation by TGF-β or epidermal growth factor (EGF) at 5 min, while reducing it at later time points. Conversely, pAkt formation by TGF-β or EGF was inhibited by CE, suggesting a direct effect on the Akt pathway. The modulation of the balance between TGF-β signaling to Smad2/3 *vs*. pAkt (by TGF-β or EGF) has potential implications for hepatic diseases and malignancies.

## Abstract (web summary)

Cholesterol affects the balance between canonical Smad vs. non-canonical Akt TGF-β-induced signaling and heteromeric TGF-β receptor complexes

## Introduction

Transforming growth factor-β (TGF-β) cytokines play critical roles in multiple physiological and pathological processes, including metabolic regulation, apoptosis, inflammation, fibrosis, cancer, as well as vascular and liver diseases^1–7^. Specifically, in the liver, TGF-βs induce opposing effects in early vs. late disease stages^8^, and aberrant TGF-β signaling is involved in metabolic syndrome, hepatitis, liver fibrosis and hepatocellular carcinoma^9–11^. TGF-βs signal via heterotetrameric complexes of dual-specificity (Ser/Thr and Tyr) kinase receptors, type II (TβRII) and type I (TβRI); the latter is activin-like kinase 5 (ALK5) in most cells, including hepatocytes, or activin-like kinase 1 (ALK1) mainly in endothelial cells^12–14^. Upon ligand binding, TβRII recruits and phosphorylates TβRI, activating canonical Smad2/3 (via ALK5) or Smad1/5/8 (via ALK1). They translocate to the nucleus together with Smad4 to activate or repress transcription of multiple genes^12,15–17^. TGF-β also stimulates, in a cell context-dependent manner, non-Smad pathways, such as TGF-β-activated kinase 1 (TAK1)-p38 mitogen-activated protein kinase (MAPK)/c-Jun N-terminal kinase (JNK), extracellular signal-regulated protein kinase (Erk1/2), phosphoinositide 3-kinase (PI3K)/protein kinase B (Akt), nuclear factor κ-light chain enhancer of activated B cells (NF-κB), and Rho GTPases^18–25^. The crosstalk between Smad and non-Smad TGF-β pathways is crucial for multiple responses, including epithelial-mesenchymal transition (EMT), invasion and metastasis^25–28^. This balance is regulated at different steps, including receptor oligomerization^14^, intracellular transport (internalization and degradation)^29–32^, transcription and translation^5,33^. Crystallographic studies on the soluble ectodomains of TβRII/TβRI in complex with TGF-β1 or -β3 have validated a heterotetrameric receptor structure (TβRI and TβRII dimers, together with a dimeric ligand)^34,35^. Such complexes were also identified for the full-length receptors at the surface of live cells by immunofluorescence co-patching and patch/FRAP (IgG-mediated patching of one cell surface receptor, combined with fluorescence recovery after photobleaching (FRAP) studies on the co-expressed receptor) methodologies^14,36–39^. These studies, which include the contribution of the cytoplasmic and transmembrane domains to the receptor complexes, have shown that they can form both heteromeric (type I/II) and homomeric (I/I or II/II) complexes already in the absence of ligand (preformed complexes, PFCs), with ligand binding significantly increasing heterocomplex formation (ligand-mediated complexes, LMCs).

TGF-β signaling initiation requires the formation of heteromeric TβRI/TβRII complexes and their stimulation by ligands^12,16,40,41^. While a considerable amount of information is available on TβRI/TβRII complexes, the mechanism(s) underlying the effects of cholesterol on the formation of TβRI/TβRII PFCs or LMCs and their signaling to distinct pathways (Smad2/3 vs. non-Smad) are still unclear. The importance of these effects is highlighted by reports on crosstalk between cholesterol and TGF-β in various cancers, where dichotomous effects on EMT and tumorigenesis were proposed to be mediated by different effectors^42–45^. In this context, a major fraction of TβRI was found in detergent-resistant membranes (lipid rafts)^31,46,47^. These microdomains, of which caveolae are a subset, are enriched with cholesterol/sphingolipids and specific proteins^48–50^. The notion that TGF-β signaling is regulated by lipid rafts is supported by the inhibition of some TGF-β1-mediated non-Smad signaling pathways (Erk1/2, p38), but not Smad2/3, by cholesterol reducing drugs in HaCaT keratinocytes^47^. In hepatocytes, knockdown of caveolin-1 (Cav1) inhibited TGF-β1-induced signaling to PI3K/Akt but not to Smad2/3^51^. Signaling to PI3K/Akt by other ligands, such as epidermal growth factor (EGF) or platelet-derived growth factor (PDGF), was also reported to be affected by localization of signaling mediators to raft domains. Thus, targeting of phosphatase and tensin homolog (PTEN) to rafts inhibited phospho-Akt (pAkt) formation in response to PDGF or EGF in HeLa cells, suggested to reflect loss of pAkt-PTEN segregation^52^. On the other hand, prolonged (62 h) robust and combined reduction of cholesterol and sphingolipids inhibited Akt recruitment to the plasma membrane in T cells stimulated by antibodies to CD28 or in COS7 cells stimulated by insulin-like growth factor 1 (IGF1)^53^. Cell type dependence of the cholesterol effects is further exemplified by statin-mediated cholesterol depletion (CD) in lung epithelial cells, where the level of total Smad2/3 was increased by transcription and translation, with no effect on the ability of TGF-β to form phosphorylated Smad2/3 (pSmad2/3)^33^. Of note, deregulated cholesterol content is a defining feature of liver diseases. For example, accumulation of cholesterol, triglycerides and ceramides was observed in a murine model of hepatocellular carcinoma, and lipotoxicity mediated by overload of hepatic free cholesterol was shown to be a mechanistic driver for fibrosis in nonalcoholic steatohepatitis (NASH)^11,54^.

Despite the above data, the mechanisms underlying the effects of cholesterol on the formation of different heteromeric TGF-β receptor complexes (PFCs and LMCs), their localization to raft and non-raft domains and the effects on the balance between TGF-β signaling to distinct pathways are ambiguous. Moreover, the effects of cholesterol enrichment (CE) on these parameters are largely unexplored. Here, we investigated these issues in hepatocytes (AML12 cells) at the level of signaling initiation—i.e., the effects of cholesterol on the formation and domain localization of TβRI/TβRII complexes and the resultant alterations in the balance between TGF-β-mediated signaling to Smad2/3 vs. PI3K/Akt pathways.

## Results

### TβRII and TβRI form stable complexes in the plasma membrane of AML12 hepatocytes

TGF-β signaling is initiated by ligand stimulation of heteromeric signaling complexes comprised of TβRII and TβRI^12,16,40,41^. Despite the evidence for crosstalk between cholesterol and TGF-β in different cellular contexts^42–45^, the mechanisms underlying this connection in hepatocytes are still unclear. Here, we set to explore these mechanisms at the level of signaling initiation (TβRII/TβRI complex formation) and its modulation by the cholesterol level.

We first measured the interactions between TβRI and TβRII in the plasma membrane of untreated AML12 hepatocytes. To this end, we employed patch/FRAP^38,55,56^. In this method (for details, see “Methods”), two receptors carrying different extracellular epitope tags are coexpressed. One tagged receptor is patched and laterally immobilized by crosslinking with a double layer of IgGs, and the effects on the lateral diffusion coefficient (*D*) and the mobile fraction (*R*_f_) of the coexpressed receptor (labeled exclusively by monovalent Fab’) are measured by FRAP. The mode and extent of the interactions between the two receptors determine whether *R*_f_ or *D* of the receptor measured by FRAP are reduced. Complex lifetimes longer than the characteristic FRAP times (i.e., stable interactions) reduce *R*_f_ with no effect on *D*, since bleached Fab’-labeled receptors do not undergo appreciable dissociation from the clusters of the immobilized receptor during the FRAP measurement. On the other hand, transient interactions (short complex lifetimes) enable each Fab’-labeled receptor molecule to undergo several dissociation/association cycles from the immobilized receptors during the FRAP measurement, reducing *D* without altering *R*_f_^38,56,57^. The method also has some limitations, which include the use of overexpression of specific receptors, which we overcome in part by conducting the measurements on lower-expressing cells chosen under the microscope^38^, and the possibility that the interactions between the receptors as measured by complex formation may depend on additional proteins^14^.

The lateral diffusion of singly-expressed myc-TβRI and HA-TβRII in AML12 cells is shown in Supplementary Fig. 1. Both receptors were laterally mobile, with FRAP curves and average lateral diffusion parameters (*D* and *R*_f_) typical of transmembrane proteins (for TβRI, *D* = 2.0 × 10^−2^ μm^2^/s and *R*_f_ = 57%; for TβRII, *D* = 2.9 × 10^−2^ μm^2^/s and *R*_f_ = 62%). These values are in the same range as in the formerly-measured COS7 and several other cell types^38,58–60^. Thus, TβRI and TβRII are laterally mobile in the plasma membrane of AML12 cells. The somewhat higher *R*_f_ and *D* values of TβRII relative to TβRI are in line with earlier studies on COS7 cells^38^.

**Fig. 1:**
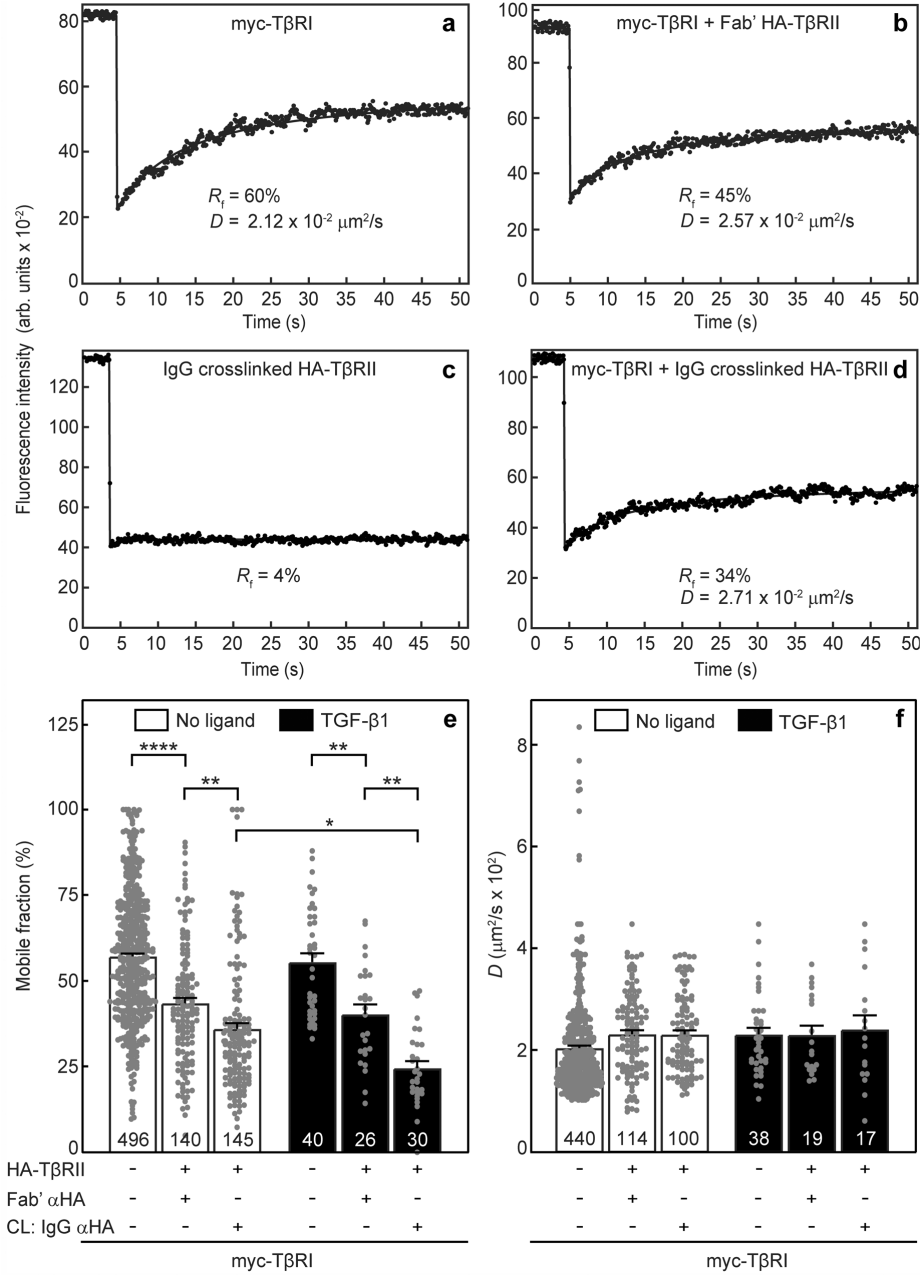
TβRI and TβRII form ligand-independent complexes which are enhanced by TGF-β1. AML12 cells were transfected with expression vectors encoding myc-TβRI, HA-TβRII, or both. When a single construct was transfected, the second one was replaced by an empty vector. After 24 h, they were serum-starved (30 min) and subjected to fluorescent labeling by monovalent Fab’ fragments (myc-TβRI) and/or IgG patching/cross-linking (CL) (HA-TβRII) as described under “Methods”. The patching/CL protocol results in HA-TβRII patched and labeled by Alexa 488-GαR IgG (designated “IgG αHA”), and myc-TβRI labeled by monovalent Fab’ (murine αmyc Fab’ followed by Alexa 546-GαM Fab’). In control experiments without crosslinking, the HA-TβRII was labeled by rabbit αHA Fab’ and Alexa 488-GαR Fab’. Where indicated, TGF-β1 (100 pM) was added during the last fluorescent labeling step, and maintained throughout the measurement. FRAP studies were conducted as described under “Methods”. Solid lines represent the best-fit of non-linear regression analysis to the lateral diffusion equation (“Methods”). Representative FRAP curves of singly-expressed myc-TβRI (**a**), myc-TβRI coexpressed with Fab’-labeled HA-TβRII (**b**), HA-TβRII immobilized by IgG crosslinking (**c**), and myc-TβRI coexpressed with IgG-crosslinked HA-TβRII (**d**). Average *R*_f_ (**e**) and *D* (**f**) values of multiple FRAP measurements. Bars depict the average values (mean ± SEM); the number of measurements (each conducted on a different cell) is shown on each bar. Some of these numbers are lower for the *D* values, since accurate *D* values cannot be derived from FRAP curves with recovery below 20%. Asterisks indicate significant differences between the *R*_f_ values of the pairs indicated by brackets (*p < 0.05; **p < 10^−3^; ****p < 10^−5^; one^-^way ANOVA and Bonferroni post hoc test). No significant differences were observed between the *D* values.

We then employed patch/FRAP to measure TβRI/TβRII complex formation in untreated AML12 cells. The cells were transfected with expression vectors for myc-TβRI and HA-TβRII (alone or together) under conditions yielding similar cell-surface levels (“Methods”), and the effects of HA-TβRII coexpression and IgG crosslinking on the lateral diffusion of myc-TβRI were measured without or with TGF-β1. It should be noted that if one of the receptors is expressed at the cell surface at significantly higher levels than its counterpart, the percentage of complexes formed would be limited by the lower-expressed receptor. TGF-β1 was added where indicated at 100 pM, based on dose-dependence analysis of pSmad2/3 formation in AML12 cells (Supplementary Fig. 2). Representative FRAP curves are shown for the lateral diffusion of singly-expressed myc-TβRI (Fig. 1a), myc-TβRI co-expressed with uncrosslinked (Fig. 1b) or IgG-crosslinked (Fig. 1d) HA-TβRII, and for the immobilization of HA-TβRII by IgG crosslinking (Fig. 1c). The average values (mean ± SEM) of multiple measurements for each condition, without and with ligand, are depicted in Fig. 1e, f. Already in the absence of ligand, *R*_f_ of myc-TβRI was significantly reduced upon coexpression with uncrosslinked HA-TβRII (from 57 to 43%; the two leftmost bars in Fig. 1e). Since the *D* value was unaffected, this may reflect the formation of stable TβRI/TβRII complexes that interact preferentially with laterally immobile cellular structures or complexes. In view of earlier reports that such immobilization could be induced by the membrane-underlying actin cytoskeleton^58,61–64^, we tested the effects of treatment of the cells with latrunculin B, an actin polymerization inhibitor^65^. This treatment increased *R*_f_ of singly-expressed myc-TβRI, and eliminated most of the reduction in its *R*_f_ when coexpressed with HA-TβRII (Supplementary Fig. 3). Thus, at least part of the immobile fraction of TβRI (alone or in complex with TβRII) are due to interactions with the cytoskeleton. Of note, immobilization of the entire HA-TβRII cell-surface population by IgG induced a further significant decrease (from 43 to 36%) in *R*_f_ of coexpressed myc-TβRI without affecting the *D* values (compare the three leftmost bars in Fig. 1e, f). Such an effect (reduction in *R*_f_ with no effect on *D*) indicates stable interactions between the two receptors on the FRAP time scale^38,56,57^, suggesting that even prior to ligand binding, stable TβRI/TβRII complexes (PFCs) may form to some degree. To validate that the mobility-restricting effects of IgG cross-linking are specific and do not involve non-specific steric trapping^66^, we probed whether the mobility of an unrelated myc-tagged receptor (ACVR2B, an activin type II receptor), is restricted by crosslinking HA-TβRII; as shown in Supplementary Fig. 4, coexpression with HA-TβRII either without or with IgG crosslinking of the latter had no effect on the lateral diffusion of myc-ACVR2B. In yet another control, we tested whether the low level of the endogenous receptors (which are not detected by Patch/FRAP but could still participate in the complexes) interferes with the measurements. As shown in Supplementary Fig. 5, siRNA knockdown of endogenous *TGFBR1* (the TβRI gene) had no significant effect on the interactions between myc-TβRI and HA-TβRII.

Addition of TGF-β1 enhanced the reduction in *R*_f_ of myc-TβRI following immobilization of HA-TβRII (Fig. 1e, rightmost white and black bars), with no change in *D* (Fig. 1f), demonstrating that the stable interactions between TβRI and TβRII are enhanced by ligand binding.

### TβRI/TβRII complexes are disrupted by cholesterol depletion and reform following ligand binding

**Fig. 2:**
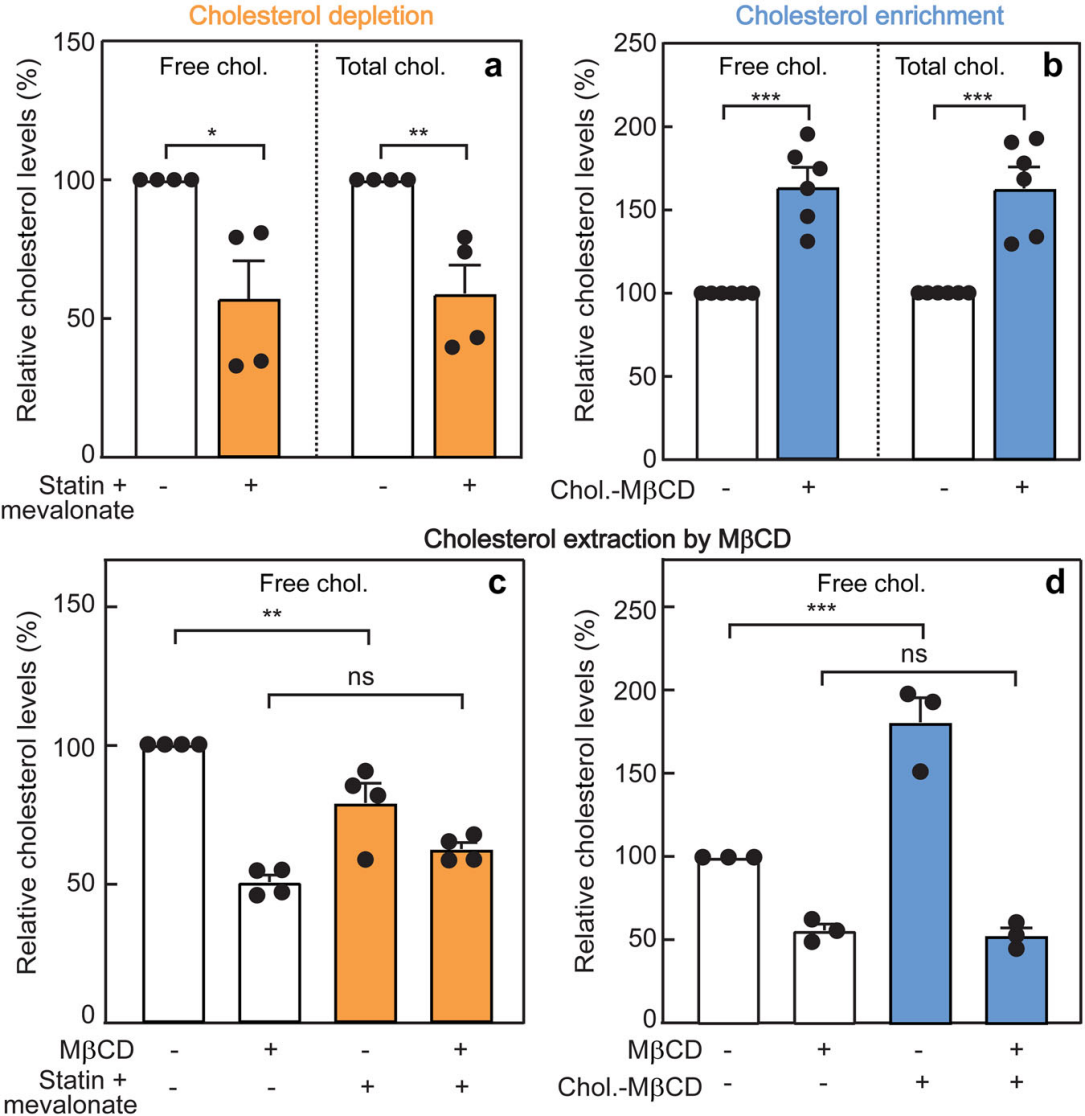
Effect of cholesterol depletion or enhancement on the cholesterol content of AML12 cells. The cholesterol (chol.) level in AML12 cells was reduced (cholesterol depletion; CD) by treatment with lovastatin and mevalonate (50 μM each) in medium containing 10% LPDS (for control samples, 10% FCS), or elevated (cholesterol enrichment; CE) by incubation with cholesterol-MβCD complex (5 mM MβCD, 300 μg/ml cholesterol) in complete growth medium for 16 h, followed by 30 min serum starvation under the same conditions (see “Methods”). The level of free (non-esterified) and total cholesterol was determined using the Abcam cholesterol assay kit (“Methods”). In each experiment, the values obtained in untreated (control) cells were taken as 100%. **a** Cholesterol depletion. Data are mean ± SEM of four independent experiments. **b** Cholesterol enrichment. Bars are mean ± SEM of six independent experiments. The results show similar levels of reduction (**a**) or elevation (**b**) in total and free cholesterol. **c** Effect of statin-mediated CD on free cholesterol level at the plasma membrane. After the CD treatment, the membrane cholesterol was extracted by short incubation with a high level of MβCD (30 mM, 30 min, 37 °C), and the free cholesterol level was assayed. Data are mean ± SEM of four independent experiments. **d** Effect of CE on the free plasma membrane cholesterol. The CE treatment was followed by extraction of the membrane cholesterol by a short exposure to MβCD as described in (**c**). Data are mean ± SEM of three independent experiments. Asterisks indicate significant differences between the pairs indicated by the brackets. ns not significant. In (**a**, **b**), significance was calculated using Student’s two-tailed *t* test. In (**c**, **d**), significance was evaluated using one-way ANOVA followed by Bonferroni post hoc test (*p < 0.05, **p < 0.01, ***p < 0.001).

To investigate the effect of CD on TβRI/TβRII heteromeric complex formation, we reduced the cholesterol level in AML12 cells by treatment with lovastatin (50 μM) in the presence of 50 µM mevalonate for 16 h, followed by 30 min starvation under the same conditions (see “Methods”). This concentration of lovastatin inhibits 3-hydroxy-3-methyl-glutaryl-coenzyme A reductase (HMG-CoA reductase); however, mevalonate (the product of HMG-CoA reductase catalysis) was added at a concentration which inhibits cholesterol synthesis but is sufficient for prenylation and geranylgeranylation, which also depend on that pathway^67–70^. This treatment resulted in similar reduction of total and free (non-esterified) cholesterol (~45%; Fig. 2a) in AML12 cells. To assess whether the CD-induced decrease in free cholesterol reflects reduced level at the plasma membrane (where the interactions between the signaling TGF-β receptors are measured by patch/FRAP), we extracted the plasma membrane cholesterol pool by short incubation (30 min, 30 mM, 37 °C) with a high level of MβCD^71,72^. While significant differences in free cholesterol were observed between control and CD conditions prior to MβCD-mediated extraction (control > CD, Fig. 2c, bars 1 and 3), plasma-membrane cholesterol extraction with MβCD resulted in equally low levels of free cholesterol (Fig. 2c, bars 2 and 4). These results suggest that most (if not all) the free cholesterol reduction upon CD treatment (~25%; Fig. 2c, bars 1 and 3) is at the plasma membrane.

**Fig. 3:**
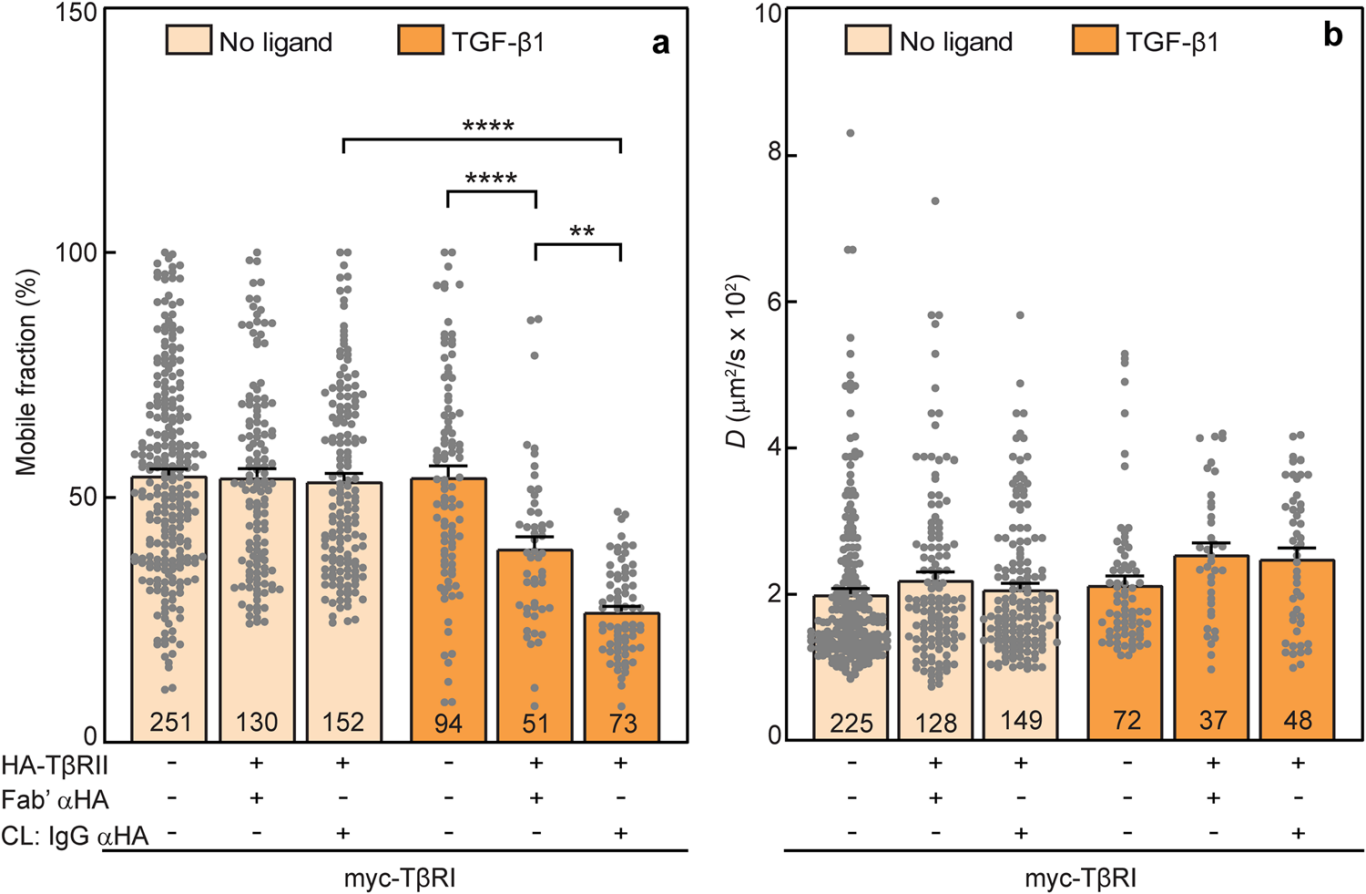
Cholesterol depletion disrupts TβRI/TβRII preformed but not ligand mediated complexes. At 6 h post-transfection with myc-TβRI alone or together with HA-TβRII as in Fig. 1, AML12 cells were subjected to CD (16 h; see “Methods”), serum-starved for 30 min, and taken for patch/FRAP studies (the patching/labeling protocol is described under “Methods” and in Fig. 1). Where shown (CL: IgG αHA), HA-TβRII was immobilized by IgG-CL. Where indicated (dark orange bars), 100 pM TGF-β1 were added during the last fluorescent antibody labeling step of the patch/FRAP protocol, and maintained throughout. The lateral mobility of Fab’-labeled myc-TβRI was measured by FRAP. **a** Average *R*_f_ values; **b** Average *D* values. Bars are mean ± SEM; the number of measurements (on different cells) is depicted within each bar. Of note, the reduction in *R*_f_ of myc-TβRI upon coexpression with HA-TβRII with or without immobilization of HA-TβRII, which was seen in untreated cells in the absence of ligand (Fig. 1e, white bars), was completely abolished by CD (**a**, light orange bars). On the other hand, addition of TGF-β1 fully restored the reduction in myc-TβRI *R*_f_ values by HA-TβRII coexpression and/or CL (**a**, dark orange bars), yielding results similar to those obtained for untreated cells in the presence of ligand (Fig. 1e, black bars). No significant differences were observed between the *D* values of myc-TβRI under all conditions (**b**). These results indicate that the TβRI/TβRII PFCs are disrupted by the CD treatment, but TGF-β1-mediated complex formation is unperturbed. Asterisks indicate significant differences between the *R*_f_ values of the pairs indicated by brackets (**p < 10^−3^; ****p < 10^−5^; one*-*way ANOVA followed by Bonferroni post hoc test).

To explore the effect of CD on the extent and dynamics of the TβRI/TβRII heteromeric interactions, AML12 cells were transfected with myc-TβRI and HA-TβRII alone or together as described in Fig. 1. At 6 h post-transfection, they were subjected (or not; control) to CD treatment (16 h), serum starved for 30 min, and taken for patch/FRAP experiments (Methods). Of note, the reduction in *R*_f_ of myc-TβRI following expression of HA-TβRII, without or with IgG crosslinking of HA-TRII, was completely abrogated by CD (Fig. 3a). Thus, in the absence of ligand (light orange bars), neither *R*_f_ nor *D* of myc-TβRI were affected (Fig. 3a, b). However, in the presence of TGF-β1 (dark orange bars), the fraction of mobile myc-TβRI was diminished by coexpressed HA-TβRII, an effect which was enhanced by HA-TβRII immobilization (Fig. 3a), while the *D* value of myc-TβRI was unaltered (Fig. 3b). These data suggest that CD disrupts the formation of TβRI/TβRII PFCs, while formation of ligand-mediated TβRI/TβRII complexes (LMCs) is unperturbed.

### TGF-β1-independent TβRI/TβRII interactions are enhanced by cholesterol enrichment

To study the effects of enhanced cholesterol levels (CE) on the interactions between TβRI and TβRII, AML12 cells transfected with myc-TβRI and/or HA-TβRII as in Fig. 3 were subjected to CE by incubation with soluble cholesterol-methyl-β-cyclodextrin (MβCD) complex (5 mM MβCD, 300 μg/ml cholesterol, 16 h) in growth medium containing 10% FCS, followed by 30 min starvation under the same conditions (“Methods”). This treatment elevated the total and free cholesterol content of the cells by 55–60% (Fig. 2b). To explore the effects of the CE treatment on the amount of cholesterol at the plasma membrane, we subjected the treated cells (vs. control cells) to cholesterol extraction from the plasma membrane by short incubation with a high MβCD concentration (30 mM, 30 min, 37 °C). As shown in Fig. 2d, significant differences in free cholesterol were observed between CE and control cells prior to short-term MβCD treatment (~80% CE > control; Fig. 2d, bars 1 and 3). In sharp contrast, MβCD extraction of plasma membrane cholesterol resulted in equally low levels of free cholesterol in CE and control cells (Fig. 2d, bars 2 and 4), demonstrating that most of the enrichment in cholesterol by CE is at the plasma membrane.

**Fig. 4:**
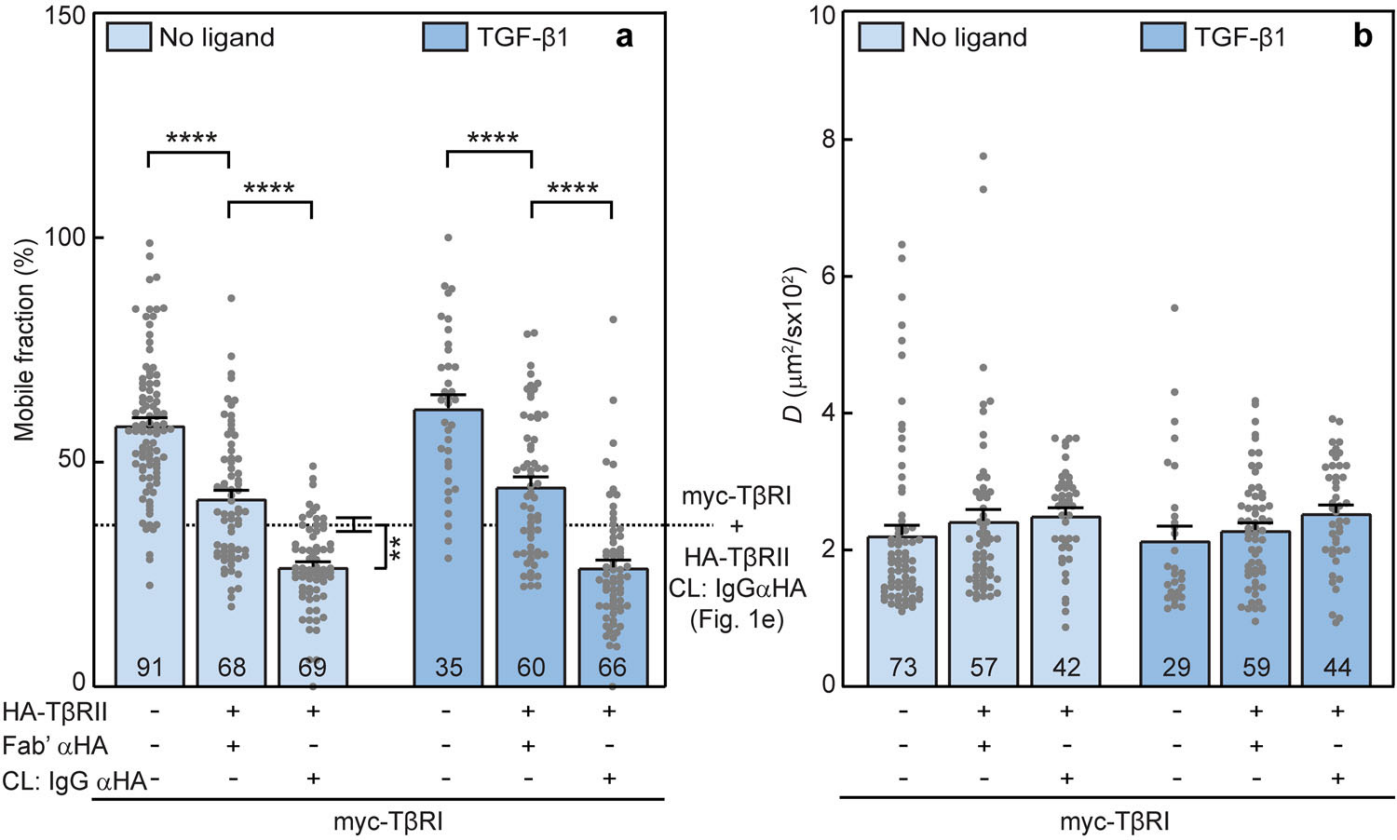
Cholesterol enrichment enhances TβRI/TβRII preformed complexes. AML12 cells were transfected with myc-TβRI alone or together with HA-TβRII. They were then labeled with fluorescent Fab’ and/or IgG and subjected to patch/FRAP studies exactly as in Fig. 3, except that the cholesterol-altering treatment employed was CE (5 mM MβCD complexed with 300 μg/ml cholesterol, 16 h; see “Methods”). CL: IgG αHA designates experiments where HA-TβRII was immobilized by IgG. Patch/FRAP experiments were conducted either without ligand (light blue bars) or with 100 pM TGF-β1 (dark blue bars), added during the last fluorescent antibody labeling step and maintained throughout. The lateral mobility of Fab’-labeled myc-TβRI was measured by FRAP. **a** Average *R*_f_ values; **b** Average *D* values. Bars are mean ± SEM; the number of measurements (each conducted on a different cell) is given within the bars. The dotted line in (**a**) depicts *R*_f_ obtained for myc-TβRI coexpressed with IgG crosslinked HA-TβRII in untreated cells (taken from Fig. 1e, third bar from the left, as indicated in the margin to the right). The further reduction in *R*_f_ of myc-TβRI upon CE (third light blue bar in **a**) suggests that CE enhances the formation of TβRI/TβRII PFCs. These stronger interactions could mask additional incremental changes in *R*_f_ in the presence of ligand, as suggested by the similar differences between the *R*_f_ values obtained upon incubation with TGF-β1 (**a**, dark blue bars). No significant differences were found between *D* values of myc-TβRI under all conditions (**b**). Asterisks indicate significant differences between the *R*_f_ values of the pairs indicated by brackets (**p < 10^−3^; ****p < 10^−5^; one*-*way ANOVA followed by Bonferroni post hoc test).

After the serum starvation, the cells were labeled with fluorescent Fab’ and/or IgG as in Fig. 3. Patch/FRAP studies conducted on these cells (Fig. 4) demonstrate that unlike CD, CE treatment enhanced TβRI/TβRII interactions in the absence of ligand (light blue bars). This is indicated by the significantly stronger reduction in *R*_f_ of myc-TβRI following immobilization of HA-TβRII as compared to untreated cells (Fig. 4a, light blue bars; the dotted line displays the value in untreated cells). The *D* values were not affected (Fig. 4b), suggesting that the TβRI/TβRII PFCs are stable on the FRAP timescale. Incubation with TGF-β1 did not induce further reduction in *R*_f_ of myc-TβRI following coexpression with HA-TβRII (without or with IgG crosslinking), most likely due to the already strong reduction in these *R*_f_ values following CE, which could mask further incremental reduction in *R*_f_. These findings indicate that elevation of the cellular cholesterol level enhances the formation of TβRI/TβRII PFCs, with no observable effect on the ligand-mediated TβRI/TβRII complexes.

### TGF-β signaling to Smad2/3 is modulated but not disrupted by cholesterol-altering treatments

**Fig. 5:**
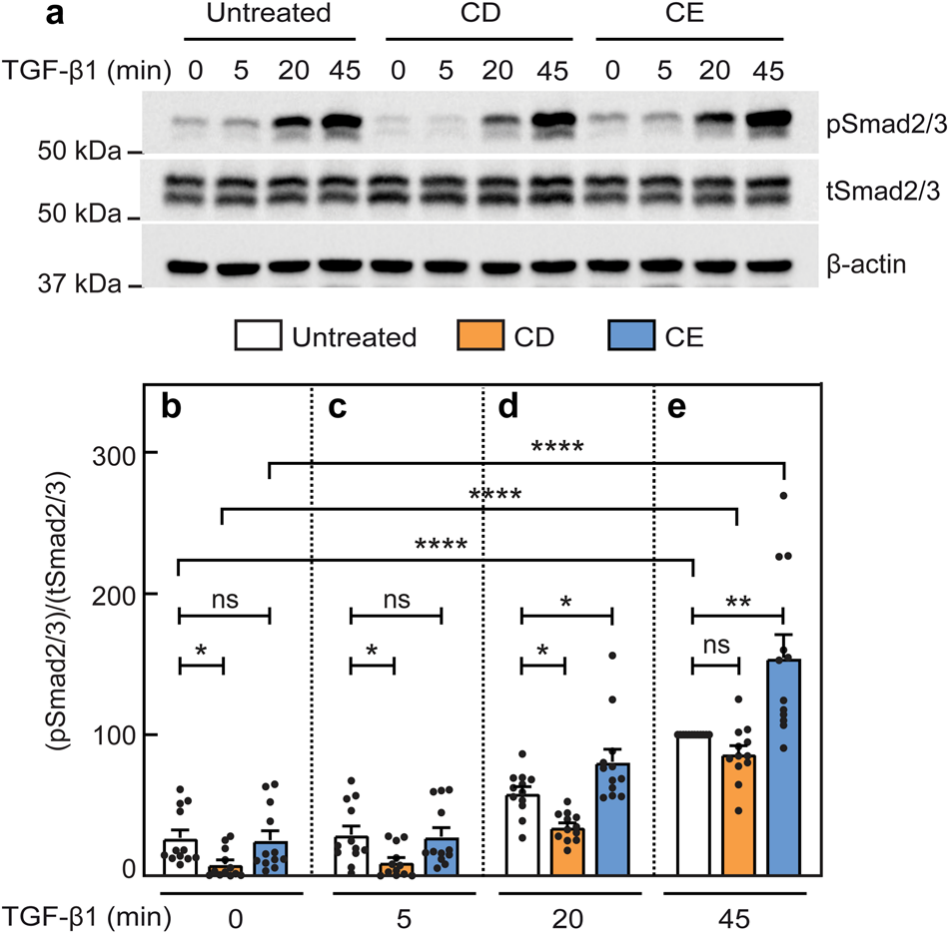
Cholesterol depletion reduces basal pSmad2/3, while cholesterol enrichment enhances TGF-β1-mediated pSmad2/3 formation at long stimulation times. AML12 cells were subjected (or not; control) to cholesterol depletion (CD) or enrichment (CE) treatments for 12 h as detailed under cholesterol treatments for signaling studies (“Methods”). After treatment, they were serum-starved (16 h) in the same medium used for the specific treatment (with 0.5% FCS for control or CE, or 0.5% LPDS for CD). The cells were then incubated without or with TGF-β1 (100 pM) for the indicated times, lysed, and analyzed by immunoblotting for phospho Smad2/3 (pSmad2/3), total Smad2/3 (tSmad2/3) and β-actin. The bands were quantified by ECL and densitometry. **a** A representative blot of pSmad2/3 formation in AML12 cells without (zero time) and with stimulation by 100 pM TGF-β1 for the indicated times. **b**–**e** Quantification of TGF-β1 signaling to pSmad2/3 relative to tSmad2/3. **b**–**e** depict stimulation for 0, 5, 20 and 45 min. Data are mean ± SEM of 12 independent experiments. The value obtained for TGF-β1 stimulation of untreated cells at 45 min was taken as 100. Asterisks indicate significant differences between the indicated pairs, using one-way ANOVA followed by Bonferroni post hoc test. Comparison of the different treatments at the same time points demonstrated a significant reduction in the basal (time 0) pSmad2/3 levels following CD, but not CE (**b**). This reduction was still noticeable at 5 and 20 min (**c**, **d**; *p < 0.05). On the other hand, CE treatment did not lead to significant differences from untreated cells at 0 or 5 min, but enhanced pSmad2/3 at 20 and 45 min (**d**, **e**; *p < 0.05, **p < 0.01). Of note, comparison of each specific treatment at different time points (using ANOVA on all bars together to compare all the white bars, orange bars, and blue bars at different time points) demonstrated significant stimulation at 20 and 45 min under all conditions (for simplicity, only the statistics for the 45 min point are shown; ****p < 10^−5^). ns not significant.

The differential effects of CD and CE on the formation of TβRI/TβRII complexes without ligand (PFCs), but not with ligand (LMCs), raise the intriguing possibility that CD and CE may alter the balance between Smad and specific non-Smad TGF-β signaling pathways. Therefore, we first investigated the effects of CD and CE on TGF-β1 signaling to Smad2/3 in AML12 cells. To this end, we employed various incubation times with 100 pM TGF-β1 (identical to the concentration used in the patch/FRAP studies). For signaling studies, AML12 cells were subjected to CD or CE (or not; control) for 12 h, followed by serum starvation (16 h) as described in Methods. These CD and CE treatment conditions resulted in cholesterol levels similar to those obtained following the analogous protocols employed for the FRAP studies (16 h treatment followed by a short 30 min starvation) (Supplementary Fig. 6). Stimulation was then initiated by adding 100 pM TGF-β1 in starvation media for the indicated times (5, 20, and 45 min). A representative experiment is shown in Fig. 5a, and quantification of multiple experiments is depicted in Fig. 5b–e. Under control conditions (no cholesterol-altering treatment), pSmad2/3 exhibited a time-dependent increase (Fig. 5b–e, white bars). Under CD conditions, significantly lower levels of pSmad2/3 were observed in the absence of ligand (Fig. 5a, b). The pSmad2/3 levels increased with the time of incubation with TGF-β1, reaching a similar level to that obtained in untreated cells at 45 min (Fig. 5a, e). Notably, at intermediate time points (5 and 20 min), the pSmad2/3 levels of CD-treated cells were significantly lower than in control cells (Fig. 5a, c, d). On the other hand, CE had no significant effect on the basal pSmad2/3 level (prior to ligand addition), as well as on the early time point post-stimulation (Fig. 5a–c). At 20 and 45 min, CE induced a moderate but significant increase in pSmad2/3 as compared to untreated cells (Fig. 5a, d, e). These findings suggest that TGF-β-mediated Smad2/3 activation persists in CD-treated cells, but is attenuated at early time points due to the reduced basal pSmad2/3 level prior to ligand addition. This is in direct correlation to the CD-mediated loss of PFCs in the patch/FRAP experiments (Fig. 3a). To validate that the CD effect on pSmad2/3 formation is a function of reduced PFCs, AML12 cells were transfected with the constitutively active myc-TβRI(T204D) mutant, whose ligand-independent activity does not depend on interaction with TβRII, together with a luciferase reporter construct and pRL-TK (Renilla luciferase, serving as a transfection calibration control). This was followed by measurement of transcriptional activation of the Smad2/3 pathway by the luciferase activity of the (CAGA)_12_-Luc reporter^29,73^, without TGF-β stimulation. Similar levels of the myc-TβRI(T204D) at the cell surface in untreated and CD-treated transfected cells were validated by measuring the fluorescence intensity in a point-confocal spot on the cell surface, as described for the endocytosis measurements and in Supplementary Fig. 9. As shown (Supplementary Fig. 7), transfection with myc-TβRI(T204D) induced Smad transcriptional activation, which was not affected significantly by CD.

In view of the controversy on the role of TGF-β receptor endocytosis in signaling, where contradictory effects of clathrin-mediated endocytosis and caveolar-like internalization were reported^29–32,47,51,74^, we tested whether CD or CE alter the endocytosis of TβRI. As shown in Supplementary Fig. 8, CD or CE had no significant effect on TβRI internalization, excluding endocytosis as a major contributor to the effects of CD or CE in the current studies. Moreover, the level of both TβRI and TβRII at the cell surface is not significantly altered by either CD or CE (Supplementary Fig. 9).

### Cholesterol depletion accelerates the loss of pAkt, while cholesterol enrichment inhibits pAkt formation

**Fig. 6:**
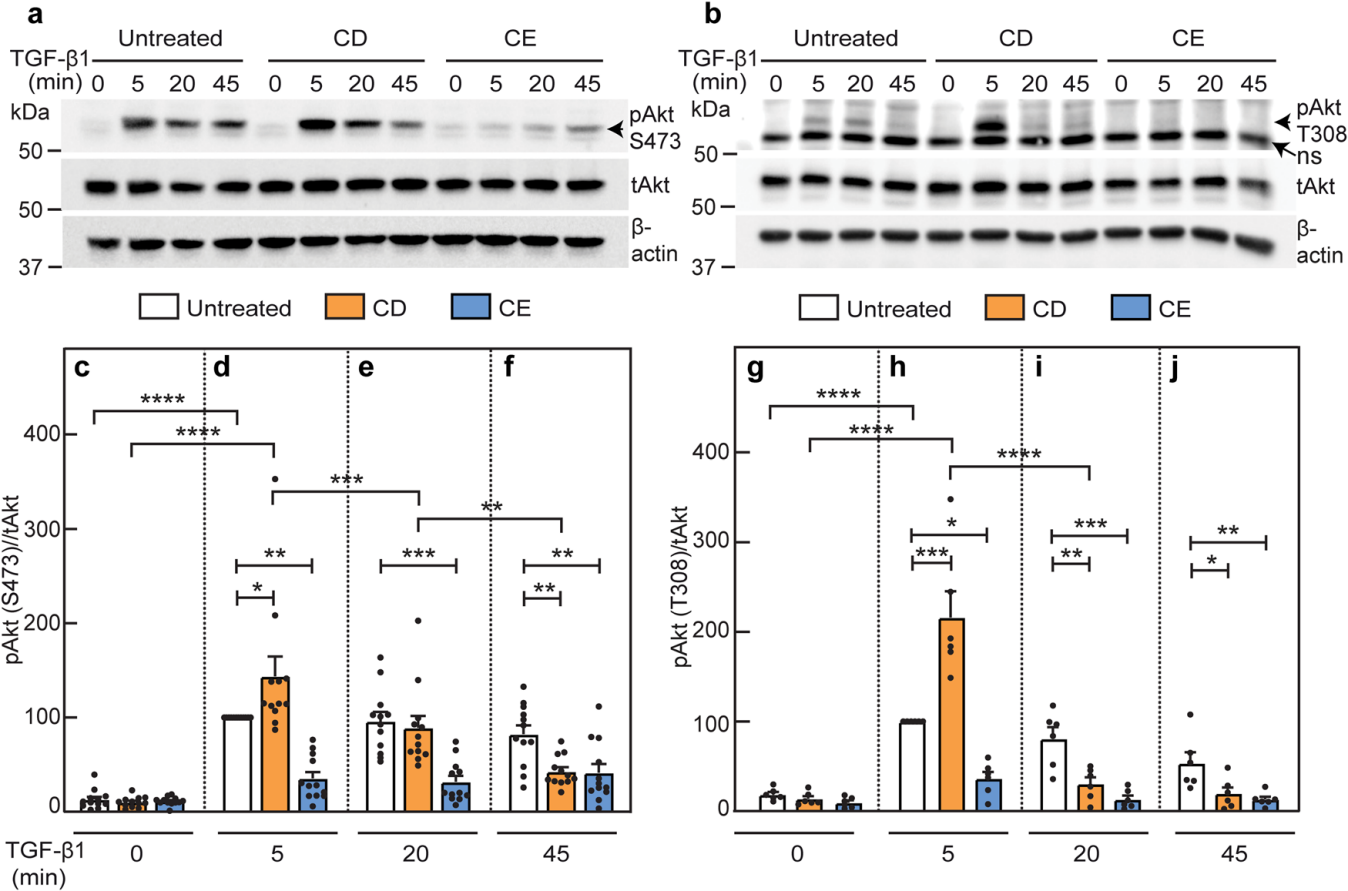
Cholesterol depletion enhances TGF-β1-mediated pAkt formation at 5 min and accelerates its loss at longer times, whereas cholesterol enrichment inhibits pAkt formation. AML12 cells were subjected (or not; control) to CD or CE followed by serum starvation exactly as in Fig. 5. The cells were then incubated ± TGF-β1 (100 pM) for the indicated times, lysed, and analyzed by immunoblotting for pAkt (pSer473 or pThr308), total Akt (tAkt) and β-actin. The bands were quantified by ECL and densitometry. **a**, **b** Representative blots of pAkt (pS473) formation (**a**) and of pAkt (pT308) formation (**b**). ns non specific band. **c**–**f** Quantification of TGF-β1 signaling to pAkt (pS473) relative to tAkt. **c**–**f** depict stimulation for 0, 5, 20 and 45 min. Data are mean ± SEM of 12 independent experiments. The values obtained for TGF-β1 stimulation of untreated cells at 5 min were set to 100. **g**–**j** Quantification of TGF-β1 signaling to pAkt (pT308) relative to tAkt. Data are mean ± SEM of six independent experiments. pT308 levels of TGF-β1 stimulated untreated cells at 5 min were set to 100. Asterisks indicate significant differences between the indicated pairs, using one-way ANOVA followed by Bonferroni post hoc test. Comparison between the different treatments at the same time points demonstrated a significant increase in pAkt following CD at 5 min (**d**, **h**; *p < 0.05; ***p < 0.001), followed by accelerated loss at 20 and/or 45 min (compare orange bars in **d** with **e** and **f**, and in **h** with **i** and **j**; **p < 0.01, ***p < 0.001; ****p < 10^−5^). Conversely, CE treatment led to inhibition of pAkt formation at all time points (compare blue bars in **c** with **d**–**f**, and in **g** with **h**–**j**; *p < 0.05; **p < 0.01, ***p < 0.001). Comparison of each specific treatment at different time points (using ANOVA on all bars together, as in Fig. 5) demonstrated a high TGF-β-mediated phosphorylation of Akt on pS473 at 5 min, with lower but still significant stimulation at 20 min for untreated or CD-treated (but not CE-treated) cells (for simplicity, only the statistics for the 5 min point are shown; ****p < 10^−5^). Similar comparison for Akt phosphorylation on pT308 showed also for untreated cells a high level of pT308 at 5 min, which was lower but still significant at 20 min. In CD-treated cells, only the time point at 5 min was significant due to faster loss of pT308, which was evident at 20 min. CE treatment abrogated the TGF-β-mediated pT308 at all time points, as in the case of pS473. For simplicity, only the statistics for the 5 min points are shown; ****p < 10^−5^).

Smad and non-Smad pathways were proposed to be affected by localization of TGF-β receptors in cholesterol-sensitive domains^31,46,47^. Therefore, we next studied the effects of CD and CE on TGF-β1-mediated activation of Akt (pAkt formation) in AML12 cells. CD, CE and TGF-β1 stimulation were conducted as described for the studies on Smad2/3 phosphorylation. In cells with unperturbed cholesterol levels, TGF-β1 induced maximal Akt phosphorylation (both at Ser473 and Thr308) at 5 min, remaining relatively stable up to 45 min for Ser473 and reduced with time for Thr308 (Fig. 6; white bars). CD treatment not only did not interfere with the ability of TGF-β1 to induce pAkt (either pSer473 or pThr308) at 5 min, but even increased it. However, it induced a faster and more prominent reduction in the pAkt levels at later times (Fig. 6). In sharp contrast, CE dramatically reduced TGF-β-induced pAkt formation at all time points (Fig. 6, blue bars). Together, these data demonstrate that alterations in the cholesterol level alter the balance between TGF-β signaling to the canonical Smad pathway vs. non-canonical pathways (here, pAkt).

**Fig. 7:**
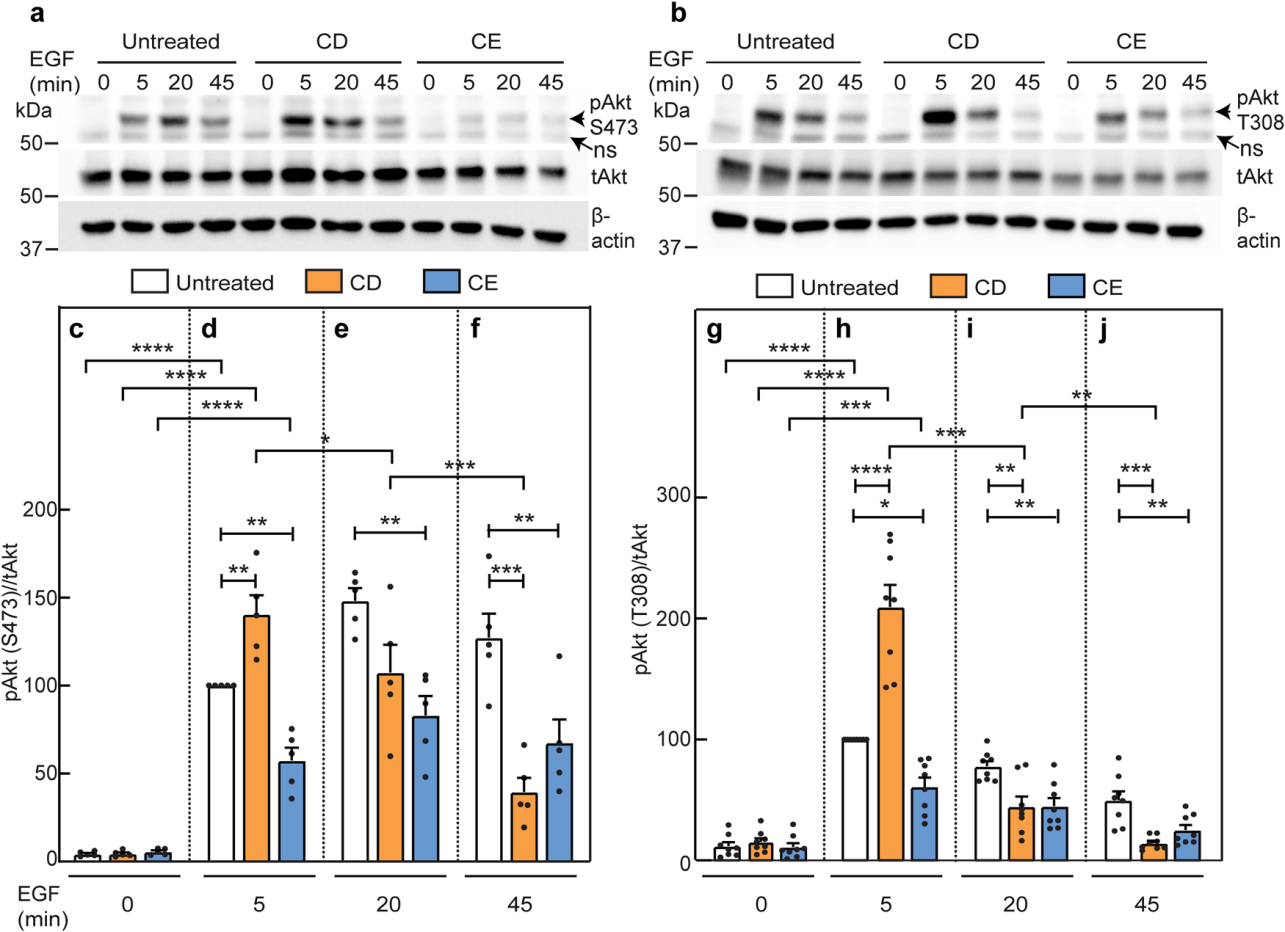
EGF-mediated pAkt formation is affected by cholesterol depletion or enrichment similar to TGF-β-induced pAkt formation. Experiments were conducted on AML12 cells as in Fig. 6, except that stimulation was with 10 nM EGF. Cells were then lysed, immunoblotted for pAkt (pSer473 or pThr308), tAkt and β-actin. Representative experiments of pAkt (pS473) formation in response to EGF (**a**) and of pAkt (pT308) formation (**b**). ns non specific band. **c**–**f** Quantification of EGF-stimulated pAkt (pS473) formation. **c**–**f** depict stimulation for 0, 5, 20 and 45 min. Data are mean ± SEM of five independent experiments. The values obtained for EGF stimulation of untreated cells at 5 min were taken as 100. **g**–**j** Quantification of EGF signaling to pAkt (pT308). Data are mean ± SEM of eight independent experiments. pT308 levels of EGF stimulated untreated cells at 5 min were set to 100. Asterisks indicate significant differences between the indicated pairs, using one-way ANOVA followed by Bonferroni post hoc test. Comparison between cholesterol treatments at the same time points showed a significant increase in pAkt (both pS473 and pT308) mediated by CD at 5 min (**d**, **h**; **p < 0.01; ****p < 10^−5^), followed by accelerated loss at 20 and/or 45 min (compare orange bars in **d** with **e** and **f**, and in **h** with **i** and **j**; *p < 0.05; **p < 0.01; ***p < 0.001). Conversely, CE treatment partially inhibited EGF-induced pAkt formation at all time points (compare blue bars in **c** with **d**–**f**, and in **g** with **h**–**j**; *p < 0.05; **p < 0.01). Comparison of each treatment at different time points (using ANOVA on all bars together) showed a high EGF-mediated pS473 formation in untreated cells at all time points. For CD-treated cells, the pS473 level was high at 5 min, becoming lower but still significant at 20 min. Analogous comparison for EGF-mediated pT308 yielded a similar pattern for both untreated cells and the effects of CD and CE at all time points. For simplicity, only the statistics for the 5 min points are shown (***p < 10^−3^; ****p < 10^−5^).

**Fig. 8:**
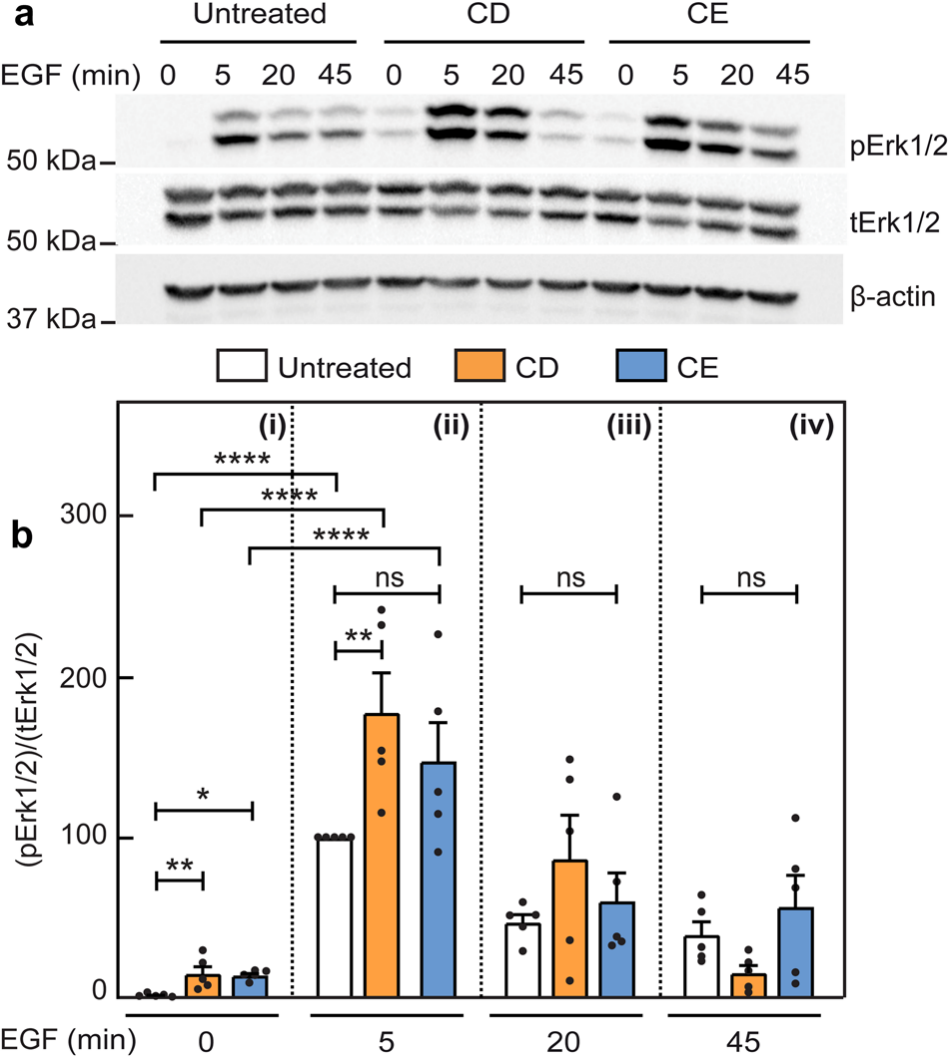
Stimulation of pErk1/2 by EGF persists following cholesterol enrichment. Studies were conducted on AML12 cells as in Fig. 7. After stimulation with EGF (10 nM), the cells were lysed and immunoblotted for pErk1/2, tErk1/2 and β-actin. **a** A representative immunoblot. **b** Quantification of EGF-stimulated pErk1/2 formation relative to tErk1/2. Panels i, ii, iii, iv depict stimulation for 0, 5, 20 and 45 min. Data are mean ± SEM of five independent experiments. The values obtained for EGF stimulation of untreated cells at 5 min were taken as 100. Asterisks indicate significant differences between the indicated pairs, using one-way ANOVA followed by Bonferroni post hoc test. Comparison of untreated, CD- and CE-treated cells at the same time points demonstrated a significant increase in the basal (time 0; unstimulated) pErk1/2 levels following either CD or CE (**b**i; *p < 0.05, **p < 0.01), and at 5 min stimulation following CD (**b**ii; **p < 0.01). Of note, CE treatment had no significant effect (ns) on EGF-mediated pErk1/2 formation at all time points.

To examine whether the effects of CD and/or CE on Akt phosphorylation apply also to other pAkt-inducing pathways, we stimulated AML12 cells subjected (or not) to the cholesterol-altering regimes with EGF (10 nM) for the indicated times. In control cells, EGF-mediated Akt phosphorylation reached a maximal value at 20 min (pSer473) or 5 min (pThr308) (Fig. 7). In contrast, in CD-treated cells, EGF-induced pAkt formation was maximal at 5 min for both phosphorylation sites, but was lost faster than in control cells (Fig. 7). Both of these observations in CD-treated cells were similar to those measured for TGF-β stimulation of pAkt. The similarity to TGF-β signaling extended also to CE, which led to partial inhibition of EGF-mediated pAkt formation at all time points (Fig. 7). This could reflect a dependence of both TGF-β and EGF signaling to pAkt on lipid rafts, or direct effects of CE on the EGF receptor. In the second case, the inhibition of EGF signaling would be expected to extend also to the canonical EGF pathway (Erk1/2 activation). However, CE had no effect on EGF-mediated Erk1/2 phosphorylation (Fig. 8), suggesting that the CE-mediated inhibition of pAkt formation is a property dependent on the Akt pathway rather than on inhibition of EGF receptor activity. Of note, CD increased pErk1/2 levels at 5 min as in the case of EGF-mediated Akt phosphorylation (Fig. 7), and both CD and CE increased the basal pErk1/2 levels, in line with earlier reports on such an effect by CD^75,76^. We conclude that the onset of Akt phosphorylation by either TGF-β or EGF persists in CD-treated cells, while the accelerated loss of pAkt at longer stimulation times may reflect faster dephosphorylation, in accord with the proposal that segregation between raft-localized pAkt and non-raft PTEN prolongs pAkt lifetime^52^. Moreover, the inhibition of pAkt formation by CE reflects a specific effect on the Akt signaling pathway.

Since CE inhibits TGF-β and EGF-mediated Akt phosphorylation both at Ser473 and Thr308, a likely possibility is that CE affects an upstream component, i.e., PI3K. We therefore explored whether CE reduces also TGF-β1 and EGF-induced phospho-PI3K (pPI3K) formation. TGF-β1 induced a 2-fold increase in pPI3K (the p55 subunit) in control cells at 5 min, remaining at this level at 20 and 45 min, while CE abrogated this effect at all time points (Supplementary Fig. 10). EGF stimulation induced a stronger effect (~10-fold) at 5 min and was reduced at 20 and 45 min, but was also effectively inhibited by CE (Supplementary Fig. 11). Thus, the effects of CE on pAkt are recapitulated by similar effects on pPI3K.

## Discussion

Lipid rafts form membrane microdomains which segregate membrane receptors and/or membrane associated signaling effectors at the cell surface, and are considered to play critical roles in the regulation of various signaling pathways^77^. Multiple studies have compared control vs. cholesterol-depleted cells for effects on signaling, whereas the effects of cholesterol enrichment, which are more relevant to disease states, have not been extensively addressed. This holds also for TGF-β receptors, which were reported to partially localize in rafts, with consequences for receptor stability, internalization, the formation of receptor oligomers and signaling^31,46,47^. Complex formation between type I and type II TGF-β receptors is essential for multiple signaling events^12,14,16,40,41^. However, the effects of cholesterol on the degree and dynamics of TGF-β receptors heteromeric complex formation, i.e., formation of PFCs and LMCs, were not fully characterized. To study these phenomena in a cell type where alterations in the cholesterol level are associated with disease, we investigated these issues in murine AML12 hepatocytes.

To initiate biophysical studies on complex formation between TβRI and TβRII in hepatocytes, we measured their lateral diffusion and mobile fractions by FRAP. Our studies (Supplementary Fig. 1) demonstrate that the lateral diffusion parameters of these receptors singly expressed in AML12 cells resemble those obtained in several other cell types^38,58–60^, and are in the same range reported for other TGF-β superfamily receptors^56,78^. Of note, similar to our earlier observation in COS7 cells^38^, coexpression of TβRII with TβRI sufficed for inducing a mild reduction in *R*_f_ of TβRI, with no effect on *D*. The lack of effect on *D* is in line with the weak dependence (logarithmic) of the lateral diffusion of transmembrane proteins on the mass of the protein embedded in the membrane^79^. Given that under these conditions (coexpression without crosslinking) both receptors are predicted to be laterally mobile, the lower *R*_f_ values indicate interactions of these receptor complexes with membrane-associated structures which are immobile on the FRAP timescale. Examples for such mobility-restricting interactors include the membrane-underlying cytoskeleton, the extracellular matrix, and clathrin-coated pits^58,61–64^. In the case of TβRI, alone or in complex with TβRII, at least part of these interactions appears to be with the actin cytoskeleton, as indicated by the increased *R*_f_ following treatment with latrunculin B (Supplementary Fig. 3). IgG-mediated immobilization of HA-TβRII in the absence of ligand induced a further significant reduction in *R*_f_ of coexpressed myc-TβRI with no effect on *D* (Fig. 1, white bars), suggesting that heteromeric TβRI/TβRII complexes (PFCs), which are stable on the FRAP timescale, form already prior to ligand binding. Moreover, addition of TGF-β1 enhanced the effect of immobilizing HA-TβRII, as reflected by the additional reduction in *R*_f_ of myc-TβRI (Fig. 1, black bars), indicative of ligand-mediated formation of heteromeric complexes (LMCs). It should be noted that although the current model for TGF-β receptor signaling is via a complex of two type I, two type II and one dimeric TGF-β ligand^14,34,35,38,39,80^, our results do not exclude the possibility of clustering with additional receptors in response to ligand binding^17^, or of further interactions of the TGF-β receptor complexes with e.g. integrins in focal adhesions^81^.

To explore whether the cholesterol level regulates the interactions between TβRI and TβRII, we altered the membrane cholesterol content by either CD (reduced cholesterol level) or CE (cholesterol enrichment). In cells with reduced cholesterol level, the effects of coexpressing HA-TβRII with myc-TβRI on the lateral diffusion of the latter, whether without or with IgG crosslinking of HA-TβRII, were completely lost (Fig. 3; similar values for all light-colored columns). This suggests that TβRI/TβRII PFCs are dependent on lipid rafts. This notion is further reinforced by the enhanced formation of such PFCs upon CE (compare the third light blue bar with the dotted line in Fig. 4a). For both treatments, the cholesterol level affected only the *R*_f_ but not the *D* values, suggesting that the PFCs are stable on the FRAP timescale. On the other hand, formation of TβRI/TβRII complexes in the presence of ligand was unaffected by either CD or CE, indicating that LMCs form independently of lipid rafts. Neither treatment affected the lateral mobility parameters of singly expressed TβRI (compare Figs. 1, 3 and 4). While this may reflect localization of TβRI only to non-raft regions, it does not appear to be the case, since CD and CE had significant effects on its *R*_f_ when coexpressed with TβRII. Alternatively, the lack of effect of cholesterol on the lateral diffusion of singly expressed TβRI may be due to stronger restrictions on its lateral diffusion imposed by interactions with non-raft membrane-associated structures, such as the membrane-underlying cytoskeleton or clathrin-coated pits^58^. Indeed, effects of cholesterol depletion on the lateral diffusion of raft-associated proteins were detected only in cases of faster-diffusing proteins, for example influenza hemagglutinin (which lacks interactions with the cytoskeleton or with coated pits) or lipid-anchored proteins such as H-Ras, N-Ras, or glycosylphosphatidylinositol (GPI)-anchored proteins^66,69,82^.

To examine whether CD or CE modulate the signaling outcome of TGF-β1 in AML12 cells, we opted to assess the effects on the canonical Smad2/3 pathway, and the Akt pathway as a representative non-canonical TGF-β stimulated pathway. Prior to stimulation by TGF-β1, a basal level of pSmad2/3 was observed in control and in CE-treated cells, but not in CD-treated cells (Fig. 5a, b). Given the absence of ligand, we interpret this as signaling by ligand-free PFCs, which are lost upon CD (Fig. 3). This is in line with the report that extracellular domain-truncated TβRI and TβRII coexpressed in Mv1Lu cells (wild type or lacking TβRI or TβRII) led to high ligand-independent receptor signaling, and coexpression of wild type TβRI and TβRII was capable of inducing a low but significant level of constitutive activity^83,84^. TGF-β1-mediated activation of Smad2/3 persisted in both CD or CE-treated cells (Fig. 5). However, at intermediate time points of TGF-β1 stimulation (5 and 20 min), the pSmad2/3 levels of CD-treated cells were significantly lower than in control cells (Fig. 5a, c, d), suggesting a contribution of PFC signaling at the early time points of Smad activation. CE-mediated enhancement of pSmad2/3 formation was observed at the later time points (20 and 45 min; Fig. 5a, d, e). As this enhancement parallels a higher level of PFCs under CE conditions, we propose that the enhanced level of TβRI/TβRII PFCs contributes to signaling to Smad2/3 at later time points. However, the possibility of contribution by CE-mediated effects which do not depend on receptor complex formation cannot be excluded. We conclude that Smad signaling proceeds under both CD and CE conditions, and can be induced by either PFCs or LMCs, both from raft and non-raft domains. This is in accordance with former studies where TGF-β1-mediated pSmad2/3 formation was reported to be relatively insensitive to cholesterol depletion or Cav1 knockdown^47,51,59^. It should be noted that CD in AML12 cells did not alter the total Smad2/3 levels, unlike their significant enhancement in Mv1Lu mink lung epithelial cells^33^, pointing to the importance of cellular context for the effects of CD.

In contrast to the relatively mild (yet significant) effects of CD and CE on Smad2/3 stimulation by TGF-β1, signaling by TGF-β1 in AML12 cells to pAkt (pSer473 and pThr308) was strongly inhibited by CE at all time points (Fig. 6). A similar phenomenon was observed for pAkt stimulation by EGF (Fig. 7), while EGF stimulation of pErk1/2 formation was unaffected (Fig. 8a, b). Thus, we propose that the effect of CE on pAkt formation reflects changes in the organization of raft domains and/or localization of Akt pathway components in these domains under CE conditions, and is due to properties of the PI3K/Akt pathways rather than TGF-β signaling. This notion is supported by the finding that CE also inhibits the phosphorylation of PI3K by TGF-β1 and EGF (Supplementary Figs. 10 and 11). The Akt pathway depends on multiple proteins, ligands and lipid targets^85,86^. Moreover, localization of different pathway components, such as 3-phosphoinositide-dependent protein kinase-1 (PDPK1) and PTEN, to distinct compartmentalized membrane microdomains was shown to be required for PI3K/Akt signaling^52^. Thus, alterations to such compartmentalization (e.g., induced by CE) may affect the signaling output independently of the ligand/receptor complexes which initiate the signaling process (here, either TGF-β1 or EGF). Further support for the involvement of segregation in lipid rafts in the regulation of Akt phosphorylation by TGF-β1 (Fig. 6) or EGF (Fig. 7) is given by the contrasting effects observed with CD-treated cells. Here, for both ligands, higher levels of pAkt were induced at 5 min stimulation as compared with control cells, demonstrating that signaling to pAkt does not require lipid rafts. Since PFCs of TGF-β receptors depend on lipid rafts, these findings, which are in contrast with the lower levels of TGF-β-induced Smad2/3 activation following CD treatment, support the notion that TGF-β-mediated activation of Akt is not dependent on PFCs. Yet, other non-Smad signaling pathways and their activation by other TGF-β superfamily receptors could still depend to some degree on raft localization, as was suggested for BMP2-mediated signaling to p38 in C2C12 cells^87^. Of note, at longer stimulation times with either TGF-β1 or EGF (Figs. 6 and 7), pAkt was lost significantly faster in CD-treated cells, in line with intermixing of raft-localized (e.g., Akt) and non-raft (e.g., PTEN) Akt signaling mediators^52^. Thus, a potential role of the PFCs is to maintain a certain balance between pSmad2/3 and the pAkt pathway (and possibly additional pathways) under basal conditions (prior to stimulation by TGF-β). Moreover, since the formation of pSmad2/3 in response to TGF-β1 is stronger in the presence of PFCs (compare untreated to CD-treated cells; Fig. 5b–e), another potential role of PFCs is to enable a faster response to TGF-β stimulation, as the PFCs are pre-formed and may therefore respond faster^14^.

**Fig. 9:**
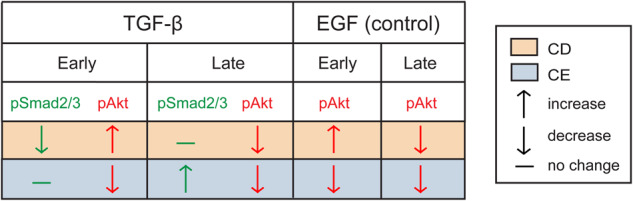
Schematic representation of the effects of cholesterol on the balance between TGF-β signaling to pSmad2/3 vs. pAkt. The effects of CD (orange) or CE (blue) on TGF-β- or EGF-mediated levels of the indicated pathways at early (5 min) or late (45 min) stimulation time points are depicted as ↑ (increase), ↓ (decrease), or − (no change). The outcomes on EGF-mediated signaling to pAkt are shown as control. For TGF-β, the balance of (pSmad2/3)/pAkt signaling is altered depending on the treatment (CD vs. CE), as well as on stimulation time. Thus, at early times, CD reduces pSmad2/3 and increases pAkt, while at late times, this effect is reversed (pSmad2/3 remains unchanged, while pAkt is reduced). CE has no effect on pSmad2/3 stimulation at early times but reduces pAkt, an effect opposite to that of CD, while at later times it retains the same effect on pAkt and raises pSmad2/3 even higher. For EGF, the effects of CD and CE on pAkt are similar to those of TGF-β.

In summary, we show that in AML12 hepatocytes, the balance between TGF-β-induced canonical signaling to Smad2/3 and non-canonical signaling to pAkt is altered differently by CD and CE (Fig. 9). For example, at 5 min of TGF-β1 stimulation, CD induces a significant reduction in pSmad2/3 due to the loss of signaling from PFCs. On the other hand, the signaling to pAkt is increased at this short stimulation time (pAkt↑, pSmad2/3↓; Figs. 5c and 6d, h). At later time points (45 min), the effect of CD on the balance between signaling to pSmad2/3 vs. pAkt is reversed (no effect on pSmad2/3, vs. pAkt↓). This may also explain the differences between the time-dependent effect on pAkt formation we observed and the contrasting reports of either lack of effect of CD on TGF-β-mediated pAkt formation or its reduction upon Cav1 knockdown, which were assessed at 30–120 min^47,51^. As for CE, inhibition of TGF-β-mediated pAkt formation alters the balance with signaling to Smad2/3 in yet another manner (pSmad2/3↑, pAkt↓, particularly noticeable at 45 min). Of note, the effects of CD and CE on pAkt are not restricted to TGF-β signaling, but remain valid also for EGF stimulation (Figs. 7 and 9). This is predicted to alter also the cellular interpretation to concomitant stimuli by TGF-β and EGF. Such concomitant stimulation under conditions of altered cholesterol may be physiologically relevant in liver diseases characterized by cholesterol overload, as well as in hepatocellular carcinoma settings^11,54^. It would therefore be of interest to explore in future studies whether similar effects are observed in vivo and in human cells and tissues.

## Methods

### Reagents

Recombinant human TGF-β1 (cat. #100-21C) was from PeproTech (Rocky Hill, NJ). Media and cell culture reagents (fetal calf serum, L-glutamine, penicillin-streptomycin were from Biological Industries (Beit Haemek, Israel). Hanks’ balanced salt solution (HBSS) with Ca^2+^/Mg^2+^ without phenol red (cat. #009015237500), 4-(2-hydroxyethyl)-1-piperazineethanesulfonic acid (HEPES, 1 M, pH 7.3; cat. #000773233100) and phosphate buffered saline (cat. #001623237500) were from Bio-Lab Ltd. (Jerusalem, Israel). Lovastatin (cat. #438185) was obtained from Merck-Calbiochem (Darmstadt, Germany). Mevalonate (as DL-mevalonic acid lactone; cat. #M4667), soluble cholesterol-methyl-β-cyclodextrin (MβCD) complex (cat. #C4951), protease inhibitor cocktail (cat. #P8340), latrunculin B (cat. #L5288) and Na_3_VO_4_ were from Sigma-Aldrich (St. Louis, MO). The cholesterol assay kit (cat #ab65359) was from Abcam (Cambridge, UK). Dual-Luciferase Reporter (DLR) assay system (cat. #E1960), including the renilla luciferase reporter pRL-TK, was from Promega (Madison, WI). Fatty acid-free bovine serum albumin (BSA) (fraction V; cat. #10-775-835-001) and 12CA5 murine monoclonal anti-influenza hemagglutinin tag (αHA) IgG (cat. #11-66-606-001) were obtained from Roche Diagnostics (Manheim, Germany). Murine monoclonal anti-myc tag (αmyc; cat. #626802) 9E10 IgG^88^ and HA.11 rabbit polyclonal IgG to the HA tag (rabbit αHA; cat. #923502) were from BioLegend (San Diego, CA). Monovalent Fab’ fragments were prepared from the 9E10 and 12CA5 IgG as described^37^. Alexa Fluor (Alexa) 488-goat anti rabbit (GαR) IgG (cat. #R37116), Alexa 546-goat anti mouse (GαM) F(ab’)_2_ (cat. #A-11018) and Alexa 488-GαR F(ab’)_2_ (cat. #A-11070) were from Invitrogen-Molecular Probes (Eugene, OR). Fluorescent F(ab′)_2_ was converted to monovalent Fab′ as described^36^. Normal goat γ-globulin (NGG; cat. #005-000-002), peroxidase-conjugated GαM (cat. #115-035-062) and GαR (cat. #111-035-144) IgGs were from Jackson ImmunoResearch Laboratories (West Grove, PA). Rabbit antibodies to phospho (p) Smad2/3 (cat. #8828), pAkt (phospho-Ser473; cat. #9271), pAkt (phospho-Thr308; cat. #9275), pPI3K [phospho-PI3K p85 (Tyr458)/p55 (Tyr199); cat. #4228), total (t) Akt (cat. #9272) and total Erk1/2 (cat. #4695S) were from Cell Signaling Technology (Danvers, MA). Rabbit antibody to total PI3K (p85) (cat. #06497) was from Upstate Biotechnology (Waltham, MA, USA). Murine monoclonal anti-pErk1/2 (diphosphorylated Erk1/2) antibody (cat. #M8159) was from Sigma-Aldrich. Murine IgG to tSmad2/3 (cat. #sc-133098) was from Santa Cruz Biotechnology (Santa Cruz, CA), and mouse anti-β-actin (cat. #08691001) from MP Biomedicals (Solon, OH).

### Cell culture, plasmid transfection and siRNA knockdown

AML12 murine hepatocyte cells (cat. #CRL-2254) from American Type Culture Collection (Manassas, VA) were grown at 37 °C, 5% CO_2_ in high-glucose Dulbecco’s modified Eagle’s medium (DMEM) supplemented with 10% fetal calf serum (FCS), penicillin, streptomycin and L-glutamine as described^89^. STR profiling and interspecies contamination test for AML12 cells was performed by the cell line authentication service from IDEXX (Kornwestheim, Germany) using the CellCheck^TM^ Mouse system that includes 19 species-specific STR markers. The cells were routinely analyzed by reverse transcriptase-PCR (RT-PCR) for mycoplasma contamination and found to be clean.

Expression vectors encoding human TβRI, its constitutively active mutant TβRI(T204D) (both in pcDNA3), or TβRII (in pcDNA1) with extracellular myc or HA epitope tags were described by us earlier^29,36,37,90^. N-terminally myc-tagged ACVR2B (cat. #LS-N12733) in pCMV3 was purchased from LSBio (Seattle, WA). The Smad transcriptional activation luciferase reporter construct (CAGA)_12_-Luc in pGL3ti^73^ was a gift from P. Knaus (Free University of Berlin, Germany). For Patch/FRAP experiments, AML12 cells were grown on glass coverslips in 6-well plates, and transfected by Lipofectamine™ 3000 (cat. #L3000001; Thermo Fisher Scientific, Waltham, MA) with different combinations of vectors encoding myc- and HA-tagged receptor constructs. The DNA amounts of the vectors (between 0.5 and 1 μg) were adjusted to yield similar cell surface expression levels of coexpressed epitope-tagged receptors, validated by quantitative measurement of the surface immunofluorescence of each receptor as described by us earlier^56,78^. The total DNA level was complemented by empty vector to 2 μg.

ON‐TARGETplus SMARTpool siRNA to murine *TGFBR1/ALK5* (non-interacting with human *TGFBR1*; cat. #L-040617-00-0005) and non-targeting siRNA pool (siScrambled; cat. #D-001810-10-05) were purchased from Dharmacon (Lafayette, CO). For siRNA knockdown, AML12 cells grown in 6-well plates were transfected by Lipofectamine™ 3000 with 50 nM siRNA alone or together with vectors encoding human myc-TβRI and HA-TβRII. The cells were incubated for 48 h to allow for siRNA knockdown.

### Cholesterol depletion and enrichment treatments

CD of AML12 cells was achieved by statin-mediated inhibition of HMG-CoA reductase, using lovastatin in the presence of mevalonate as described^33,67,68,70^. Mevalonate (the product of HMG-CoA reductase, added to prevent excessive reduction of mevalonate) was added at a level that reduces cholesterol production but is sufficient for farnesylation and geranylgeranylation to occur^91^, as indicated by the retention of the membrane localization of Ras proteins^69,70^. For FRAP, patch/FRAP and endocytosis studies, where long starvation after the cholesterol treatment is not required, AML12 cells were transfected as described above. Six h post transfection with epitope-tagged TGF-β receptors, the cells were subjected to CD by incubation for 16 h with lovastatin and mevalonate (50 µM each) in DMEM containing 10% lipoprotein deficient serum (LPDS), prepared as described earlier^33^; 10% FCS was used for control samples. For CE, the transfected cells were incubated 16 h in complete growth medium (with 10% FCS) supplemented with cholesterol-MβCD complex (5 mM MβCD, 300 μg/ml cholesterol). The effects of the CD and CE treatments on the cellular cholesterol content were measured using the Abcam cholesterol assay kit (cat. #ab65359) with or without cholesterol esterase to measure the levels of total and free (membrane-associated) cholesterol, respectively. After the CD or CE treatments, the cells were serum starved (with 0.5% LPDS for depletion, or 0.5% FCS for control and enrichment treatments) for 30 min, followed by the patching/labeling protocol of the cell surface receptors (see next paragraph). Where indicated, 100 pM TGF-β1 was added at the start of the incubation with antibodies (antibody labeling step) and maintained throughout the FRAP measurements.

For signaling studies, AML12 cells were treated for cholesterol depletion or enrichment exactly as above, except that the incubations (in DMEM containing 10% LPDS or FCS according to the treatment) were for 12 h. The cells were then starved for 16 h in complete DMEM containing 0.5% LPDS (for depletion) or 0.5% FCS (control or enrichment), retaining the relevant reagents (lovastatin/mevalonate or cholesterol-MβCD) in the medium. Stimulation was then initiated by adding 100 pM TGF-β1 in starvation media for the indicated times. In some control experiments, the cells were stimulated with 10 nM EGF (instead of TGF-β1) for the times indicated.

### IgG-mediated patching/crosslinking

AML12 cells plated on glass coverslips were transfected with combinations of myc-TβRI and/or HA-TβRII. Where indicated, they were subjected to cholesterol depletion or enhancement treatments as above, serum-starved (30 min, 37 °C), washed with cold HBSS supplemented with 20 mM HEPES (pH 7.4) and 2% BSA (HBSS/HEPES/BSA), and blocked with NGG (200 μg/ml, 30 min, 4 °C). For FRAP studies on singly-expressed receptors, they were then labeled successively at 4 °C (to allow exclusive cell surface labeling) in HBSS/HEPES/BSA (45 min incubations) with: (1) monovalent murine Fab’ αmyc or Fab’ of 12CA5 αHA (40 μg/ml); (2) Alexa 546-Fab’ GαM (40 μg/ml). For patch/FRAP studies, they were labeled with: (1) murine αmyc Fab’ (40 μg/ml) together with HA.11 rabbit αHA IgG (20 μg/ml); (2) Alexa 546-Fab′ GαM (40 μg/ml) together with Alexa 488-IgG GαR (20 μg/ml). This protocol results in the HA-tagged receptor crosslinked (CL) and immobilized by IgGs, whereas the myc-tagged receptor, whose lateral diffusion is then measured by FRAP, is labeled exclusively by monovalent Fab′. 100 pM TGF-β1 was added where indicated along with the labeling antibodies, and retained throughout the FRAP measurements.

### FRAP and patch/FRAP

The patch/FRAP method is described schematically in Supplementary Fig. 12. Cells expressing epitope-tagged receptors were labeled fluorescently as described above, and subjected to FRAP and patch/FRAP experiments^38,56,92^. FRAP studies were done at 15 °C, replacing samples after 20 min to minimize internalization during the measurement. An argon-ion laser beam (Innova 70C, Coherent, Santa Clara, CA) was focused through a fluorescence microscope (Axioimager.D1; Carl Zeiss MicroImaging, Jena, Germany) to a Gaussian spot of 0.77 ± 0.03 μm (Planapochromat 63x/1.4 NA oil-immersion objective) in the focal plane of the plasma membrane facing away from glass surface, choosing randomly the location of the beam on the membrane. After a brief measurement at monitoring beam intensity (528.7 nm, 1 μW), 60–75% of the fluorescence in the illuminated region was bleached (5 mW, 20 ms) and fluorescence recovery was measured by the monitoring beam. Values of *D* and *R*_*f*_ were extracted from the FRAP curves by nonlinear regression analysis, fitting to a lateral diffusion process^93^. Patch/FRAP studies were performed similarly, except that IgG-mediated crosslinking (CL) of HA-TβRII (described above) preceded the measurement^38,57,92^. Patch/FRAP measures the effects of immobilizing one receptor (here, HA-TβRII) on the lateral diffusion of co-expressed receptor (myc-TβRI, labeled by monovalent Fab’). Apart of identifying complex formation between the receptors, it distinguishes between transient and stable complexes^38,56,57^.

### Internalization measurements

AML12 cells were grown on glass coverslips and transfected with myc-TβRI as described for IgG-mediated crosslinking. Cells were either untreated, or treated for CD or CE as described under “Cholesterol depletion and enrichment treatments”. After serum starvation (30 min, 37 °C), the cells were blocked with NGG (200 μg/ml, 30 min, 4 °C), and labeled with murine αmyc IgG (20 μg/ml, 45 min, 4 °C) followed by Alexa 546-Fab’ GαM (40 μg/ml, 30 min, 4 °C), all in HBSS/Hepes/BSA. Endocytosis of myc-TβRI was quantified by the point confocal method employing the FRAP setup under non-bleaching illumination conditions^29^. Labeled cells were either fixed immediately with 4% paraformaldehyde (time 0), or warmed to 37 °C for the indicated periods to allow endocytosis, fixed (30 min at 4 °C, then 30 min at 22 °C). Endocytosis was quantified by measuring the reduction in the fluorescence intensity at the plasma membrane, focusing the laser beam through the 63x objective at defined spots (1.86 μm^2^) in the focal plane of the plasma membrane away from vesicular staining, passing the fluorescence through a pinhole in the image plane to make it a true confocal measurement.

### Western blotting

AML12 cells were grown overnight in 6-well plates, and subjected (or not; control) to the cholesterol depletion or enrichment treatments for signaling studies (see cholesterol treatments section). After 12 h of the appropriate treatment, cells were starved (16 h) in the respective media (continuing the treatment during starvation) in the presence of 0.5% LPDS (CD treatment) or 0.5% FCS (control or CE treatment). They were then stimulated (or not) with 100 pM TGF-β1 for the indicated times, washed by phosphate buffered saline and lysed on ice with RIPA lysis buffer (137 mM NaCl, 20 mM Tris-HCl, 2 mM EDTA, 0.5% SDS, 7 mM sodium deoxycholate, 1% TritonX-100, 10% glycerol, 1% protease inhibitor cocktail and 0.1 mM Na_3_VO_4_). After low-speed centrifugation, the lysates were subjected to SDS-PAGE (10% polyacrylamide) and immunoblotting as described^56^. The blots were probed (12 h, 4 °C) by rabbit anti pSmad2/3 (1:1000), murine anti tSmad2/3 (1:1000), rabbit anti pAkt or tAkt (1:1000), or mouse anti β-actin (1:50,000), followed by peroxidase-GαR or -GαM IgG (1:5000, 1 h). The bands were visualized by enhanced chemiluminescence (ECL) using Clarity ECL substrate (cat. #1705060, Bio-Rad, Hercules, CA), recorded using ChemiDoc Touch imaging system (Bio-Rad) and quantified by Image Lab software (Bio-Rad).

### Transcriptional activation assays

AML12 cells grown in 96 wells were cotransfected using Lipofectamine™ 3000 with: (1) 20 ng luciferase reporter construct (CAGA)_12_-Luc; (2) 15 ng pRL-TK (Renilla luciferase construct); (3) 100 ng empty vector or a vector encoding myc-TβRI(T204D). At 24 h post-transfection, the cells were treated for CD (or left untreated) for 12 h as described under “Western blotting”, starved (16 h) in the respective media (continuing the treatment during starvation) in the presence of 0.5% LPDS (CD treatment) or 0.5% FCS (control). The cells were lysed, and analyzed by the DLR assay system. The results were normalized for transfection efficiency using the Renilla luminescence.

### Real-time quantitative reverse transcriptase-PCR (RT-qPCR)

AML12 cells were subjected to siRNA transfection for 48 h. Total RNA was isolated using TRIzol^®^ reagent (cat. #15596026; Invitrogen) according to the manufacturer’s instructions. RNA was reverse transcribed to cDNA using qPCRBIO cDNA Synthesis kit (cat. #PB30.11, PCR Biosystems, London, UK). The mRNA levels of endogenous *TGFBR1* were determined in triplicate by RT-qPCR using Applied Biosystems Fast SYBR™ Green Master Mix (cat. AB-4385612, Thermo Fisher Scientific), and quantified with Applied Biosystems 7300 Real-Time PCR System Software (Thermo Fisher Scientific). Relative mRNA expression values were calculated based on the comparative threshold cycle (C_T_) method^94^, normalizing the data to *TUBB2a*. The sequences of the primers (Sigma) used were: (1) *TGFBR1* forward primer—CCTTGAGTCACTGGGTGTTATG; reverse primer—CCACTTAGCTGTCACCCTAATC. (2) *TUBB2a* forward primer—CATCAGCAAGATCAGAGAGGAG; reverse primer—CACTGAGAGGGTGGCATTATAG.

### Statistics and reproducibility

Statistical analysis was done by Prism9 (GraphPad Software, San Diego, CA). Significant differences between multiple data sets were evaluated by one-way ANOVA followed by post hoc Bonferroni test. Student’s *t* test was used to calculate the significance of the difference between two groups. Data are presented throughout as mean ± SEM, along with the number of independent measurements (given within each figure or figure legend). *p* values below 0.05 were defined as statistically significant.

### Reporting summary

Further information on research design is available in the Nature Portfolio Reporting Summary linked to this article.

### Supplementary information

Supplementary information

Description of Additional Supplementary Files

Supplementary Data

Reporting Summary

## Data Availability

All data generated or analyzed during this study are included in this article and its Supplementary Information files. The Supplementary Information file contains all supplementary figures (Supplementary Figs. 1–12) and the original uncropped Western blots (Supplementary Fig. 13). The source data behind all graphs in the manuscript are in the Supplementary Data file.
