# Supplementary material for: Cholesterol modulates type I/II TGF-β receptor complexes and alters the balance between Smad and Akt signaling in hepatocytes
Source: Commun Biol. 2024 Jan 2;7:8. doi: 10.1038/s42003-023-05654-9 (PMC10761706; doi:10.1038/s42003-023-05654-9)

SUPPLEMENTARY INFORMATION

Supplementary Figures 1 – 13, including original uncropped Western blots

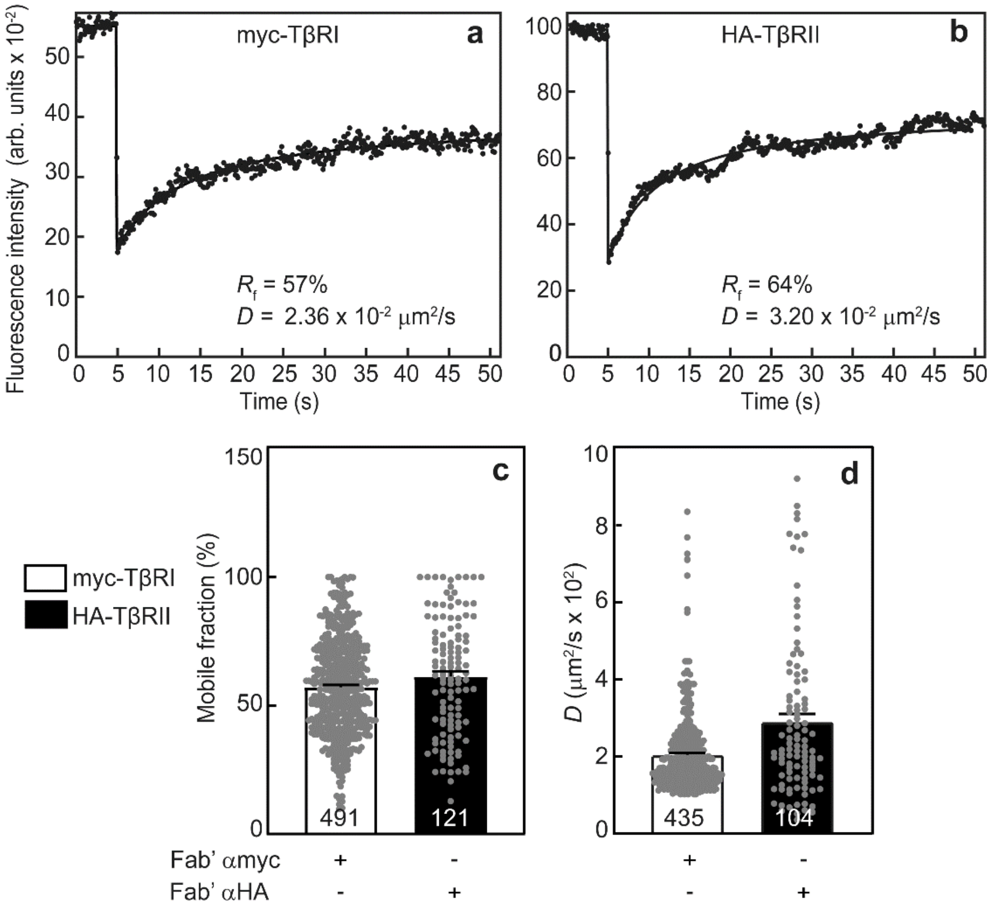

Supplementary Fig. 1 Lateral diffusion of TβRI and TβRII in the plasma membrane of AML12 cells. AML12 cells were transfected with either myc-TβRI or HA-TβRII. At 24 h post transfection, live cells were labeled at 4 °C as described under Methods, by successive incubations with (i) Fab' of murine monoclonal 9E10 αmyc for the myc-tagged receptors, or of murine monoclonal 12CA5 αHA for the HA-tagged receptor; (ii) Alexa 546-GαM Fab'. FRAP experiments were conducted as described under Methods. **a, b** Representative FRAP curves showing the lateral diffusion of singly-expressed (Fab' labeled) myc-TβRI (a) or HA-TβRII (b). Solid lines represent the best-fit of non-linear regression analysis to the lateral diffusion equation (see Methods). The R_f and *D* values of the specific curves are shown underneath each curve. **c, d** Average R_f (c) and *D* values (d) derived from multiple patch/FRAP measurements. Bars are mean ± SEM; the number of measurements for each condition is depicted within each bar. Note that curves with very low mobile fractions were taken for the R_f measurement but not for the *D* measurement (since the *D* value in such curves cannot be determined accurately).

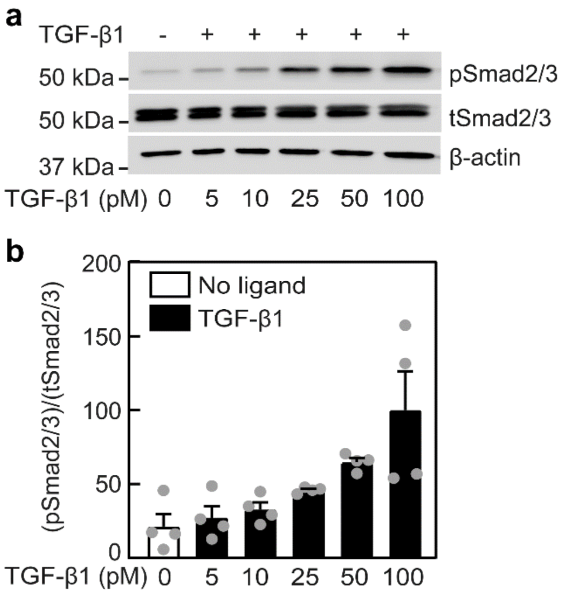

Supplementary Fig. 2 Dose-dependence of Smad2/3 activation by TGF-β1 in AML12 cells. AML12 cells were grown, starved (16 h, 0.5% FCS) and stimulated (30 min, 37 °C) with the indicated concentrations of TGF-β1 (0 pM TGF-β1 indicates unstimulated cells) as described under Western blotting (Methods). They were then lysed, subjected to SDS-PAGE and immunoblotted for pSmad2/3, tSmad2/3 and β-actin, followed by visualization of the bands by ECL and densitometry (Methods). **a** Typical immunoblots of AML12 cells stimulated by the indicated TGF-β1 concentrations for 30 min. **b** Quantification of TGF-β1 signaling to pSmad2/3 relative to tSmad2/3. Data are mean ± SEM (4 independent experiments).

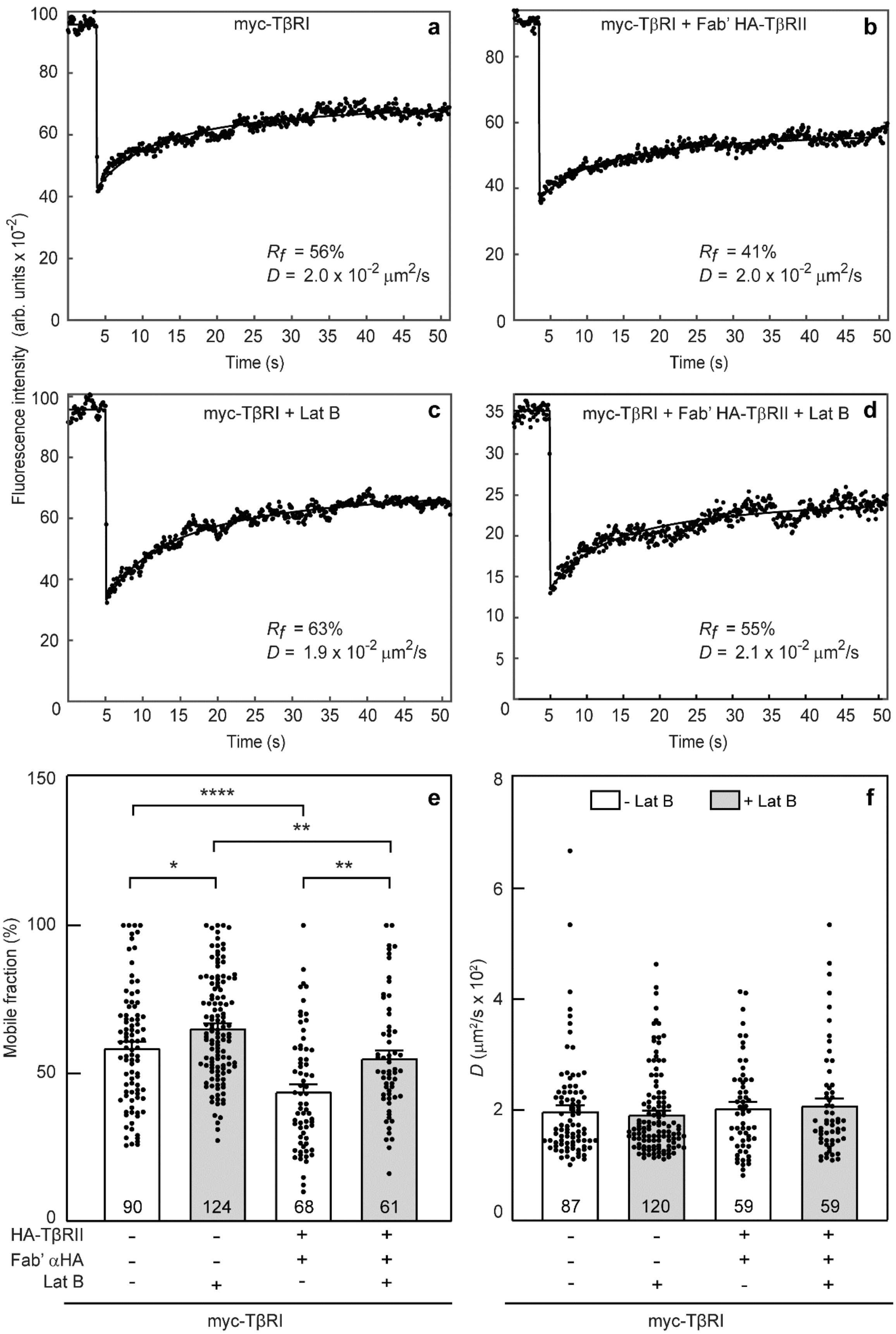

myc-TβRI
a
Rf = 56%
D = 2.0 x 10^-2 μm^2/s
myc-TβRI + Fab' HA-TβRII
b
Rf = 41%
D = 2.0 x 10^-2 μm^2/s
myc-TβRI + Lat B
c
Rf = 63%
D = 1.9 x 10^-2 μm^2/s
myc-TβRI + Fab' HA-TβRII + Lat B
d
Rf = 55%
D = 2.1 x 10^-2 μm^2/s
Fluorescence intensity (arb. units x 10^-2)
Time (s)
e
Mobile fraction (%)
90
124
68
61
HA-TβRII
Fab' αHA
Lat B
myc-TβRI
f
- Lat B
+ Lat B
D (μm²/s x 10²)
87
120
59
59
myc-TβRI

Supplementary Fig. 3 Latrunculin B treatment increases the mobile fraction of myc-TβRI. AML12 cells were transfected with expression vectors encoding myc-TβRI alone or together with HA-TβRII as in Fig. 1. After 24 h, they were serum-starved (30 min) and subjected to fluorescent labeling by monovalent Fab' (murine αmyc Fab' followed by Alexa 546-GαM Fab' for singly-expressed myc-TβRI, together with rabbit αHA Fab' and Alexa 488-GαR Fab' for cells coexpressing myc-TβRI and HA-TβRII). Latrunculin B (Lat B) treatment (1 μM for 5 min, reduced to 0.1 μM up to the end of the experiment)[65] was initiated 5 min prior to the FRAP measurements, conducted at 15 °C for up to 20 min from the start of Lat B treatment. **(a-d)** Representative FRAP curves of myc-TβRI (expressed singly or together with HA-TβRII) with or without Lat B as depicted within the panel. **(e, f)** Average R_f (e) and D values (f) of multiple experiments under each condition; the number of measurements is indicated within the bars. Bars, mean ± SEM. Asterisks mark significant differences between the R_f values of the pairs indicated by brackets (*, $p < 5\times10^{-2}$; **, $p < 10^{-3}$; ****, $p < 10^{-5}$; one-way ANOVA followed by Bonferroni post-hoc test). No significant differences were observed between the D values.

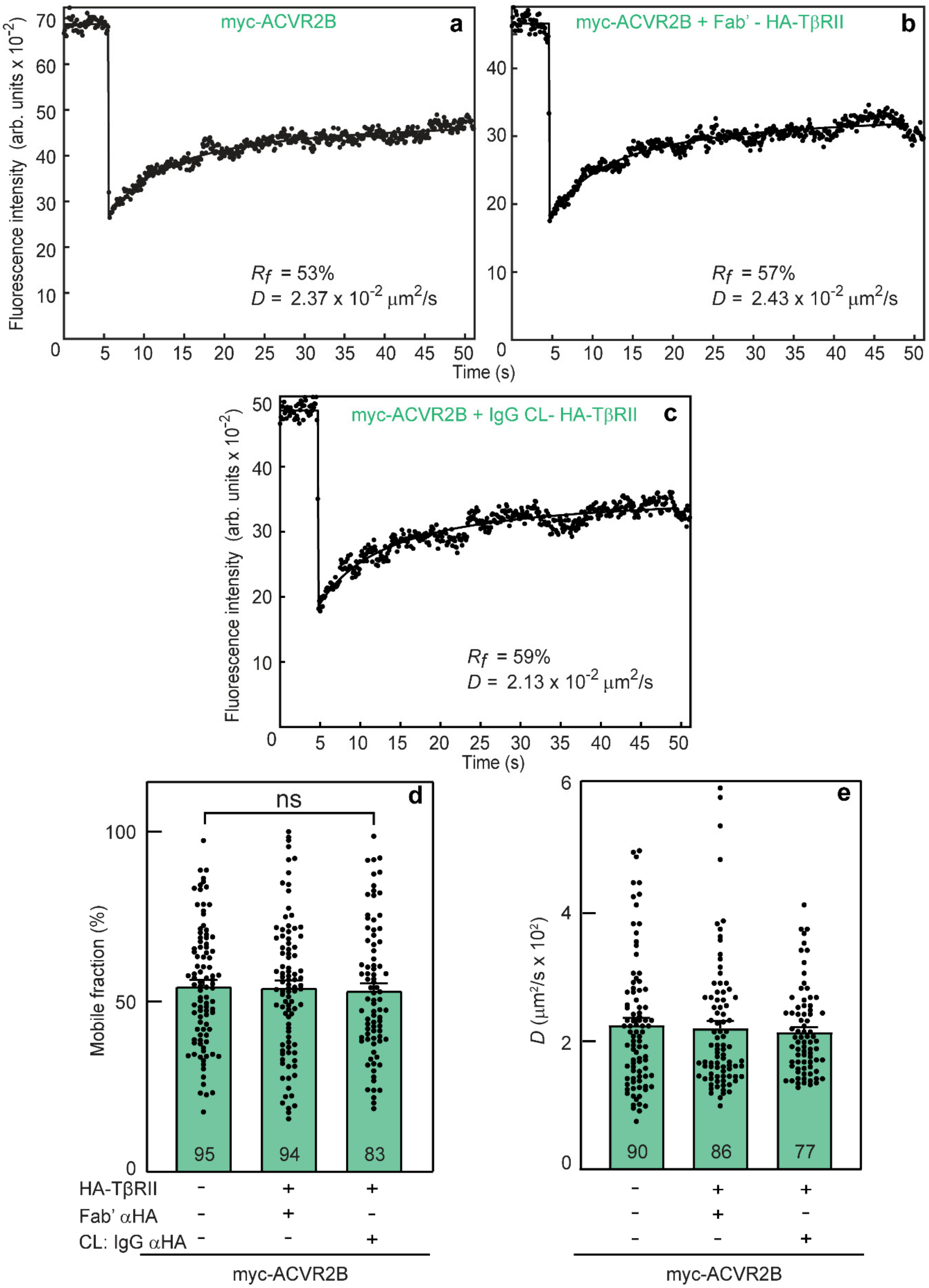

Supplementary Fig. 4 Patch/FRAP studies demonstrate that the mobility of an unrelated myc-tagged receptor is unaffected by crosslinking of coexpressed HA-TβRII. AML12 cells were

transfected with myc-ACVR2B alone or together with HA-TβRII. After 24 h, cells were subjected to IgG-crosslinking (CL) of HA-TβRII and Fab' labeling of myc-ACVR2B as in Fig. 1. In control experiments without CL, the IgG αHA was replaced by Fab' αHA. FRAP studies to measure the lateral diffusion of myc-ACVR2B were conducted as in Fig. 1. **(a-c)** Representative FRAP curves of singly expressed myc-ACVR2B (a) or myc-ACVR2B coexpressed with HA-TβRII without (b) or with (c) IgG CL. **d, e** Average R_f (d) and D values (e) demonstrate that coexpression with HA-TβRII, without or with IgG CL, does not affect R_f or D of myc-ACVR2B. Bars, average values (mean ± SEM); the number of measurements (each conducted on a different cell) is shown within the bars. No significant differences (ns) were detected between the R_f or D values (one-way ANOVA and Bonferroni post-hoc test).

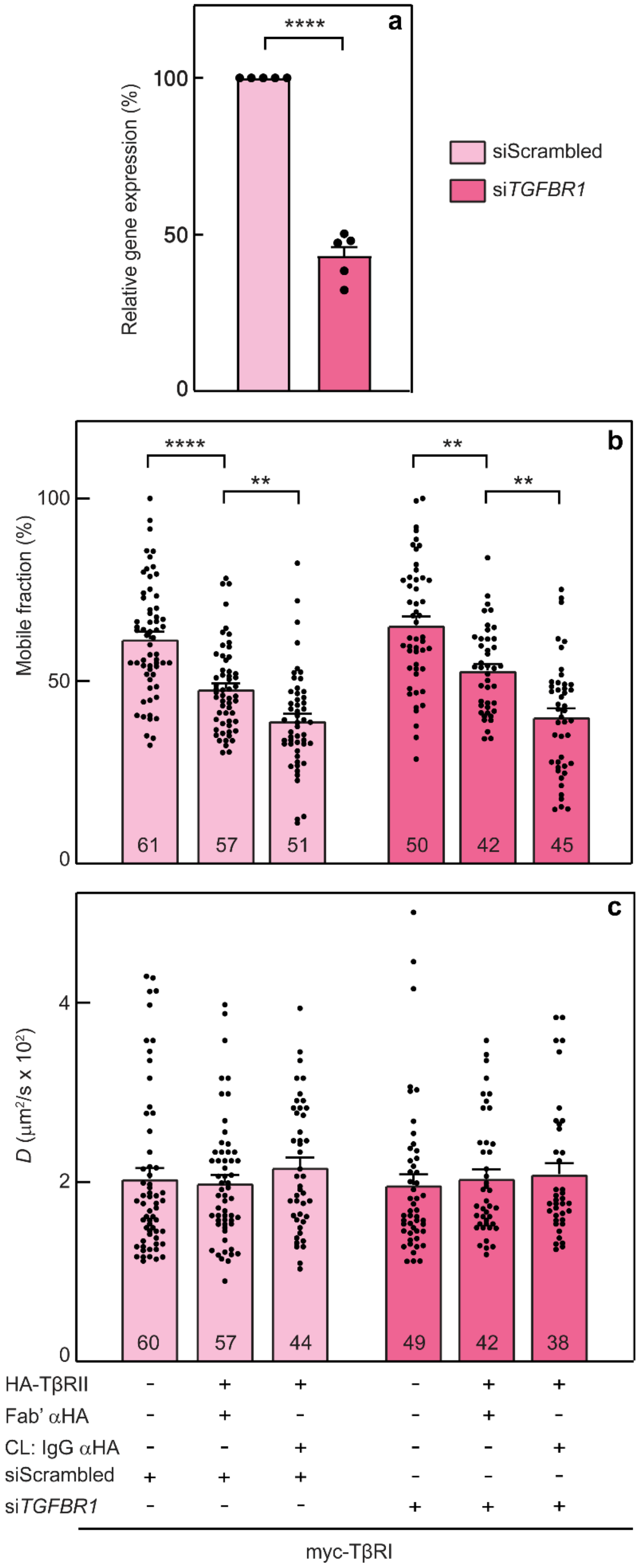
a
b
c
****
**
Relative gene expression (%)
Mobile fraction (%)
D (μm²/s x 10²)
siScrambled
siTGFBR1
HA-TβRII
Fab' αHA
CL: IgG αHA
myc-TβRI
61
57
51
50
42
45
60
44
49
38

Supplementary Fig. 5 siRNA knockdown of endogenous *TGFBR1* in AML12 cells does not affect myc-TβRI/HA-TβRII interactions. Cells were transfected with siRNA to *TGFBR1* or scrambled siRNA (control), alone (a) or together with vectors encoding human myc-TβRI and HA-TβRII (b, c). After 48 h, they were serum starved (30 min) and taken either for RT-qPCR determination of the mRNA level of *TGFBR1* (a) or for measurement of the interactions between myc-TβRI and HA-TβRII by patch/FRAP as described in Fig. 1 (b, c). **a** RT-qPCR quantification of siRNA-mediated knockdown of *TGFBR1*. Data were normalized using *TUBB2a* as the housekeeping gene, taking the level of the respective mRNA in cells transfected with siScrambled as 1. Results are the mean ± SEM of 5 independent experiments, each conducted in triplicate. The reduction in *TGFBR1* mRNA level was highly significant (****, $p < 10^{-5}$; Student's two-tailed *t*-test). **b, c** R_f (b) and D values (c) of patch/FRAP experiments. Cells were transfected with siRNA as above, together with myc-TβRI alone or with HA-TβRII. After 48 h, cells were serum starved (30 min), followed by labeling and IgG-crosslinking (CL) of the surface receptors (CL or Fab' labeling of HA-TβRII, and Fab' labeling with another fluorophore of myc-TβRI) as in Fig. 1. In cells transfected with siScrambled (light pink), coexpression with HA-TβRII reduced significantly R_f of myc-TβRI, an effect which was enhanced by IgG CL of HA-TβRII (b). siRNA knockdown of *TGFBR1* (dark pink) had no effect and yielded similar results, indicating that the endogenous TβRI does not interfere with the interactions between the epitope-tagged receptors. Bars, average values (mean ± SEM); the number of measurements (each conducted on a different cell) is indicated within the bars. Asterisks indicate significant differences between the pairs indicated by the brackets (**, $p < 0.01$; ****, $p < 10^{-5}$; one way ANOVA followed by Bonferroni post-hoc test).

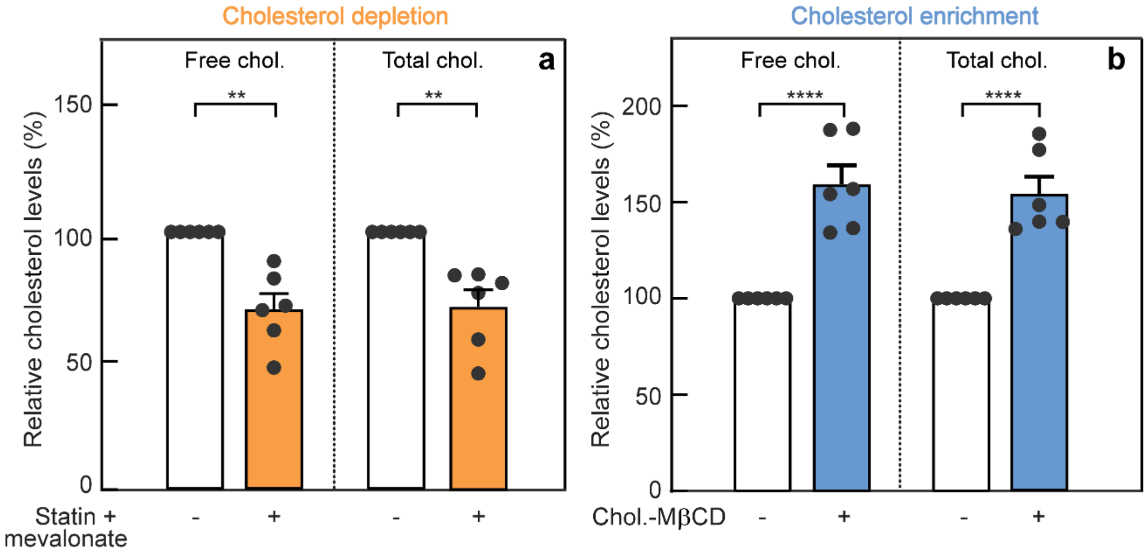

Supplementary Fig. 6 Effect of cholesterol depletion or enhancement with prolonged starvation on AML12 cholesterol content. The cholesterol (chol.) level in AML12 cells was reduced by CD treatment with lovastatin and mevalonate (50 µM each) in medium containing 10% LPDS (or 10% FCS; control). Alternatively, the cells were enriched for cholesterol (CE) by incubation with cholesterol-MβCD complex (5 mM MβCD, 300 µg/ml cholesterol) in growth medium for 12 h, followed by 16 h serum starvation under the same conditions (see Methods). The level of free and total cholesterol was determined using the Abcam cholesterol assay kit (Methods). In each experiment, the values obtained in untreated (control) cells were taken as 100%. **a** Cholesterol depletion. Data are mean ± SEM of 6 independent experiments. **b** Cholesterol enrichment. Bars are mean ± SEM of 6 independent experiments. The results show similar levels of reduction (a) or enrichment (b) in total and free cholesterol. Asterisks indicate significant differences between the pairs indicated by the brackets (Student's two-tailed *t*-test; **, $p < 0.01$; ****, $p < 10^{-5}$).

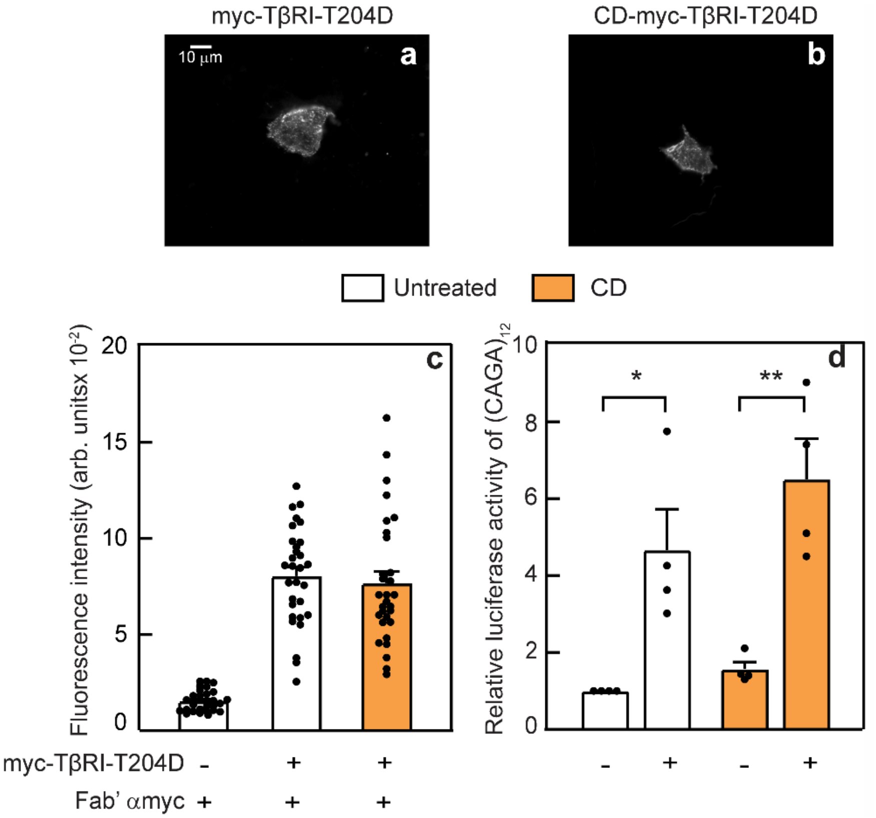

Supplementary Fig. 7 Transcriptional activation of the Smad pathway by the constitutively active myc-TβRI(T204D) mutant is unaffected by cholesterol depletion. AML12 cells were co-transfected with $(CAGA)_{12}$-Luc, pRL-TK, and myc-TβRI(T204D) or empty vector. After 24 h, they were treated for CD (or left untreated; control) for 12 h, and serum-starved (16 h). They were then subjected to measurement of the cell surface level of myc-TβRI(T204D) (**a-c**) or analyzed by the DLR assay as described under Methods (Transcriptional activation assays) (**d**). **a, b** Typical images of cells subjected to measurements of the cell-surface levels of the myc-tagged receptors, under control and CD conditions. Labeling was with a saturating concentration (40 μg/ml) of murine Fab' αmyc followed by 40 μg/ml Alexa 546-Fab' GαM; point confocal measurement of the fluorescence intensity at the cell surface was as described in Supplementary Fig. 9. **c** Quantification of the cell surface levels of multiple cells. Data are mean ± SEM of 30 measurements under each condition. No significant differences were observed between the values for untreated *vs*. CD-treated cells. **d** DLR assay of Smad2/3 transcriptional activation using $(CAGA)_{12}$. The results were normalized for transfection efficiency using Renilla luminescence. Data are presented relative to untreated, untransfected cells. Each bar is the mean ± SEM of 4 independent experiments (*, $p < 0.05$; **, $p < 0.01$; one way ANOVA followed by Bonferroni post-hoc test).

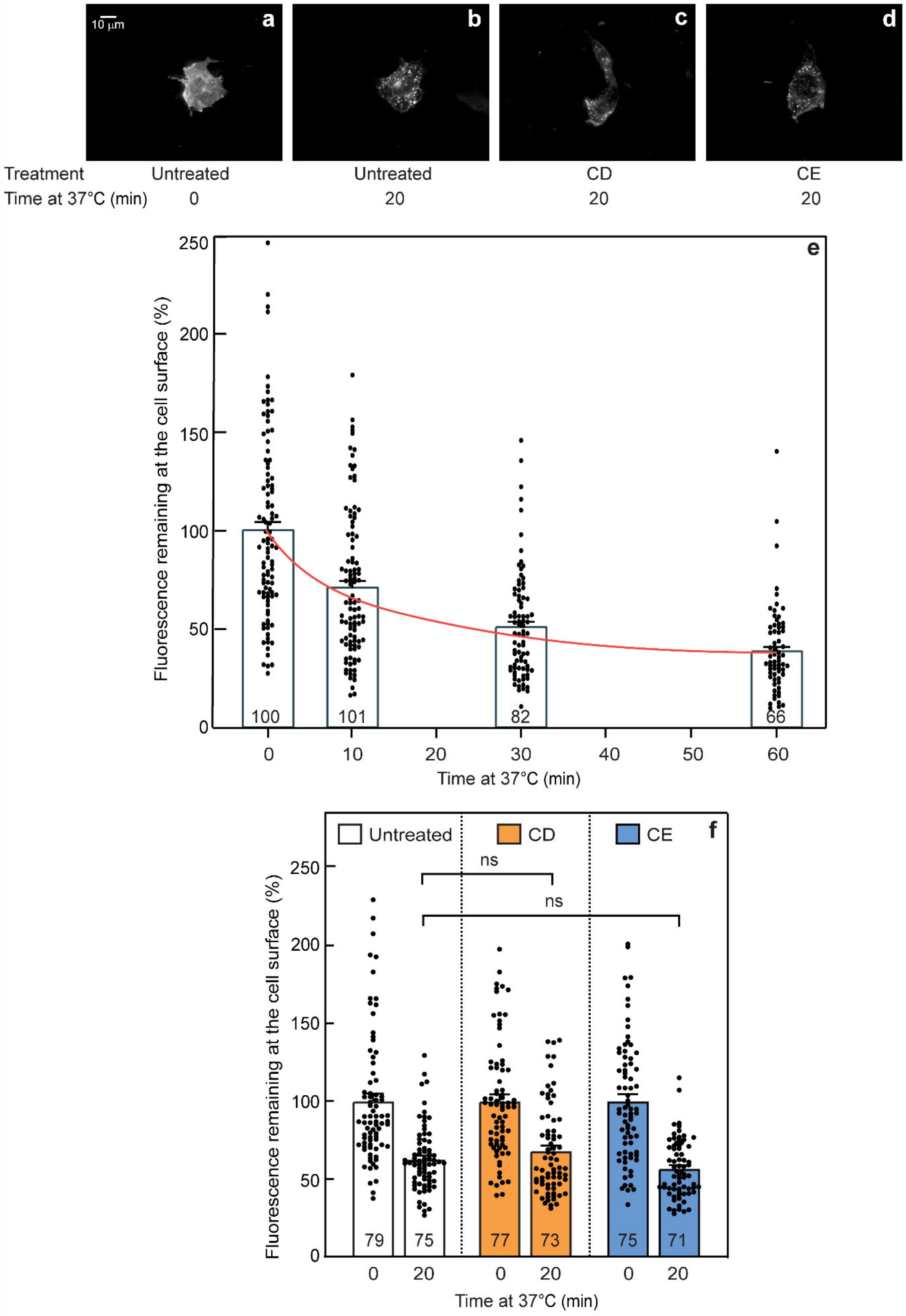
10 µm
a
b
c
d
Treatment
Untreated
Untreated
CD
CE
Time at 37°C (min)
0
20
20
20
e
Fluorescence remaining at the cell surface (%)
Time at 37°C (min)
100
101
82
66
f
Untreated
CD
CE
ns
ns
Fluorescence remaining at the cell surface (%)
Time at 37°C (min)
79
75
77
73
75
71

Supplementary Fig. 8 Endocytosis of myc-TβRI in AML12 cells is not significantly altered by CD or CE. AML12 cells were transfected with myc-TβRI. After 6 h, they were subjected (or not; control) to CD or CE (16 h) as described under Methods), serum-starved for 30 min, and taken for endocytosis measurements (Methods). **a-d** Typical images of myc-TβRI internalization at time 0 or after 20 min incubation at 37 °C. Bar, 10 μm. **e** Quantitative measurements of the endocytosis of myc-TβRI in untreated cells. The fluorescence intensity remaining at the cell surface was measured by the point confocal method (Methods). Results are mean ± SEM; the number of measurements (each conducted on a different cell) is depicted within each bar. The intensity at time zero was taken as 100%. The endocytosis half-time derived from the curve is 10.5 min, close to the 13-min value obtained for TβRI in COS7 cells[29]. **f** Effects of CD and CE on TβRI endocytosis. The experiments were as in panel e, except that the incubation time at 37 °C was 20 min (chosen to yield significant internalization), and untreated cells were compared with CD or CE-treated cells. For each treatment, the fluorescence intensity of the same sample at time zero was taken as 100%. Results are mean ± SEM; the number of measurements is indicated within each bar. No significant differences (ns) of the surface level of the receptors after 20 min endocytosis were found between treatments (one-way ANOVA followed by Bonferroni post-hoc test).

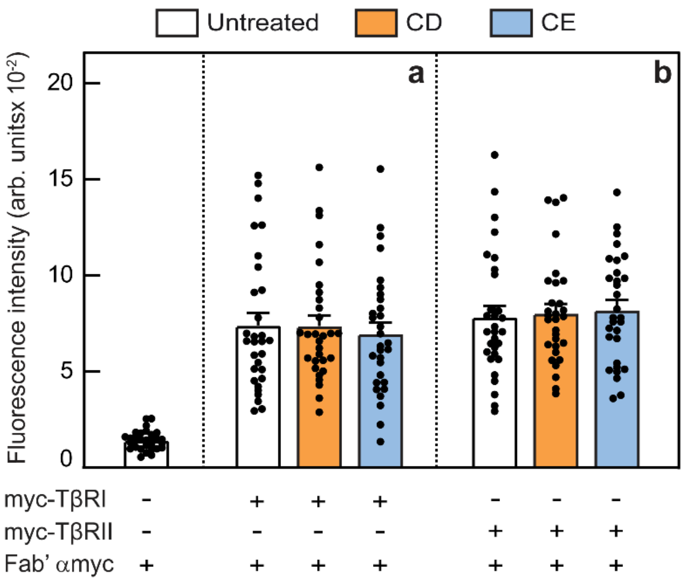

Supplementary Fig. 9 Cell surface levels of TβRI and TβRII in untreated, CD or CE-treated AML12 cells. AML12 cells were transfected with myc-TβRI or myc-TβRII, and treated (or not; control) for CD or CE followed by 30 min serum starvation as in Fig. 3. The cell surface myc-tagged receptors were labeled at 4 °C by a saturating concentration (40 µg/ml) of murine Fab' αmyc, followed by 40 µg/ml Alexa 546-Fab' GαM, and fixed (4% paraformaldehyde). This protocol enables to measure the levels of the tagged receptors at the plasma membrane under identical conditions (same laser excitation line and intensity, same microscope filters, same settings of the photomultiplier tube)[56]. The surface levels of the receptors were quantified by measuring the fluorescence intensity from a point-confocal spot by the FRAP apparatus under non-bleaching conditions as described for the endocytosis measurements. **a** Point confocal measurements of the expression levels of myc-TβRI; **b** Measurements of the surface levels of myc-TβRII. Each bar represents mean ± SEM of 30 independent measurements. No significant differences were observed between the levels of either myc-TβRI or myc-TβRII upon either CD or CE conditions (one-way ANOVA and Bonferroni post-hoc test).

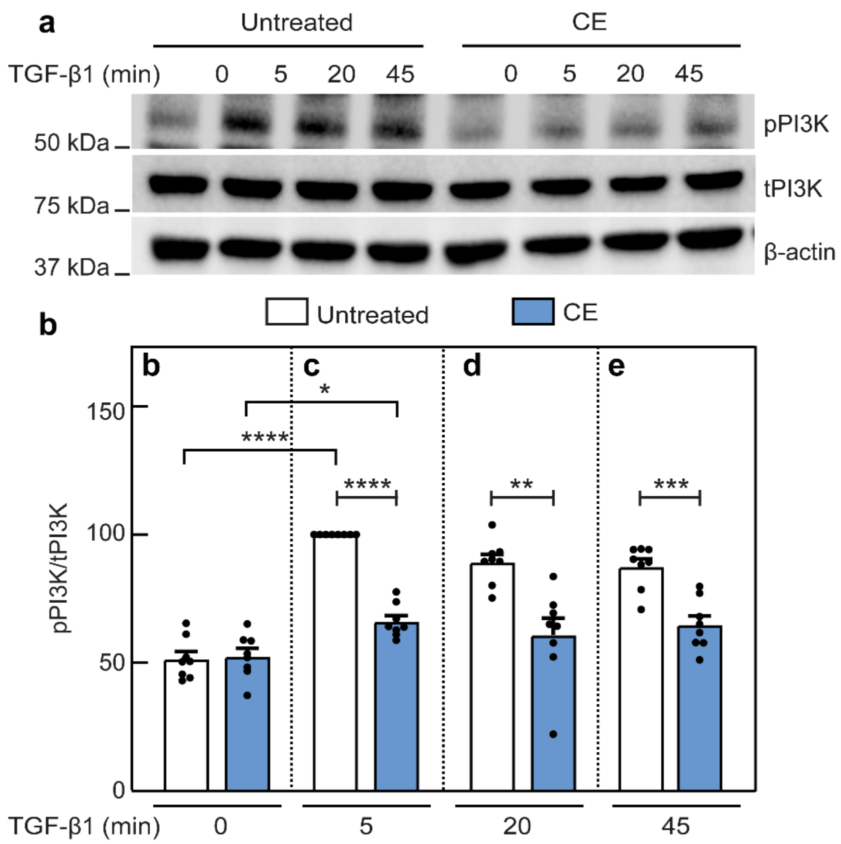

Supplementary Fig. 10 Cholesterol enrichment inhibits TGF-β1-induced pPI3K formation. AML12 cells were left untreated or subjected to CE treatment followed by serum starvation as in Fig. 5. They were then incubated without or with TGF-β1 (100 pM) for the indicated times, lysed, and analyzed by immunoblotting for pPI3K [phospho-PI3K p85 (Tyr458)/p55 (Tyr199)], total PI3K p85, β-actin. **a** A representative blot of pPI3K formation without (zero time) and with stimulation by 100 pM TGF-β1 for the indicated times. **b-e** Quantification of TGF-β1 signaling to pPI3K relative to tPI3K. Panels b, c, d, e depict stimulation for 0, 5, 20 and 45 min. Data are mean ± SEM of 8 independent experiments. The values obtained for TGF-β1 stimulation of untreated cells at 5 min were set to 100. Asterisks indicate significant differences between the indicated pairs at the same time points (Student's 2-tailed *t*-test; **, $p < 0.01$; ***, $p < 0.001$; ****, $p < 10^{-5}$). Comparison of the different time points of stimulation for untreated cells (One way ANOVA followed by Bonferroni post-hoc test on all bars together, as in Fig. 5) demonstrated a significant TGF-β-mediated stimulation of pPI3K at all time points (for simplicity, only the statistics for the 5 min point are shown. ****, $p < 10^{-5}$).

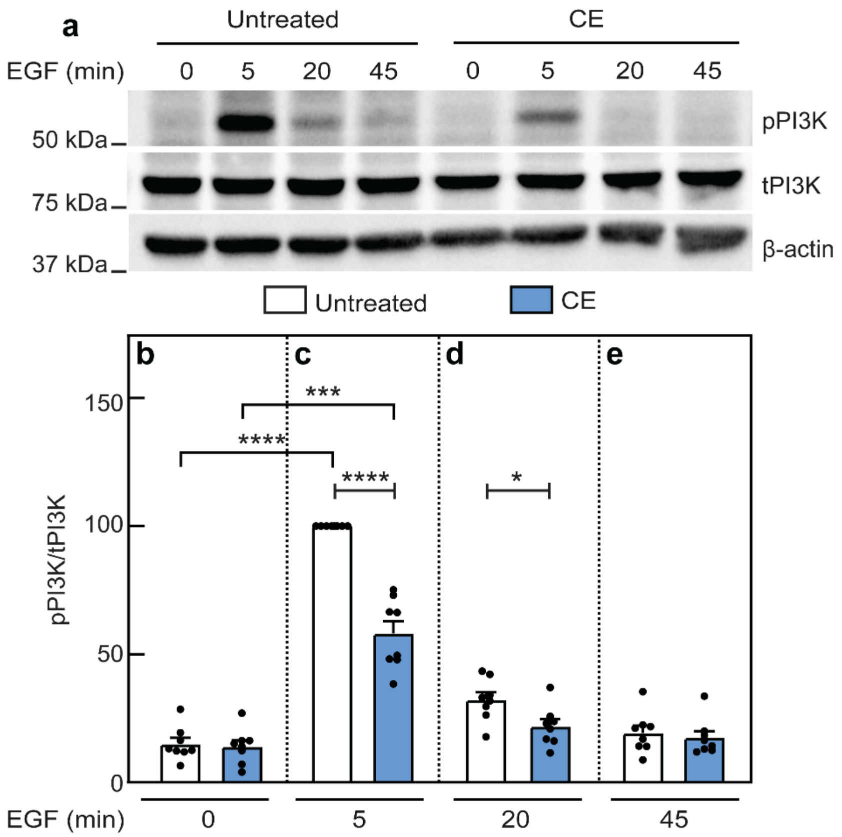

Supplementary Fig. 11 Cholesterol enrichment inhibits EGF-mediated pPI3K formation. AML12 cells (untreated or CE-treated) were serum starved and stimulated (or not) with EGF (10 nM) for the indicated times as in Supplementary Fig. 10. The cells were lysed, and analyzed by immunoblotting for pPI3K [phospho-PI3K p85 (Tyr458)/p55 (Tyr199)], total PI3K p85, β-actin. **a** A representative blot of pPI3K formation without (zero time) and with stimulation by 10 nM EGF for the indicated times. **b-e** Quantification of EGF signaling to pPI3K relative to tPI3K. Panels b, c, d, e depict stimulation for 0, 5, 20 and 45 min. Data are mean ± SEM of 8 independent experiments. The values obtained for EGF stimulation of untreated cells at 5 min were set to 100. Asterisks indicate significant differences between the indicated pairs at the same time points (Student's 2-tailed *t*-test; *, $p < 0.05$; ****, $p < 10^{-5}$). Comparison of the different time points of stimulation for untreated and CE-treated cells (one way ANOVA followed by Bonferroni post-hoc test on all bars together) demonstrated a highly significant EGF-mediated stimulation of pPI3K at 5 min (***, $p < 0.001$; ****, $p < 10^{-5}$).

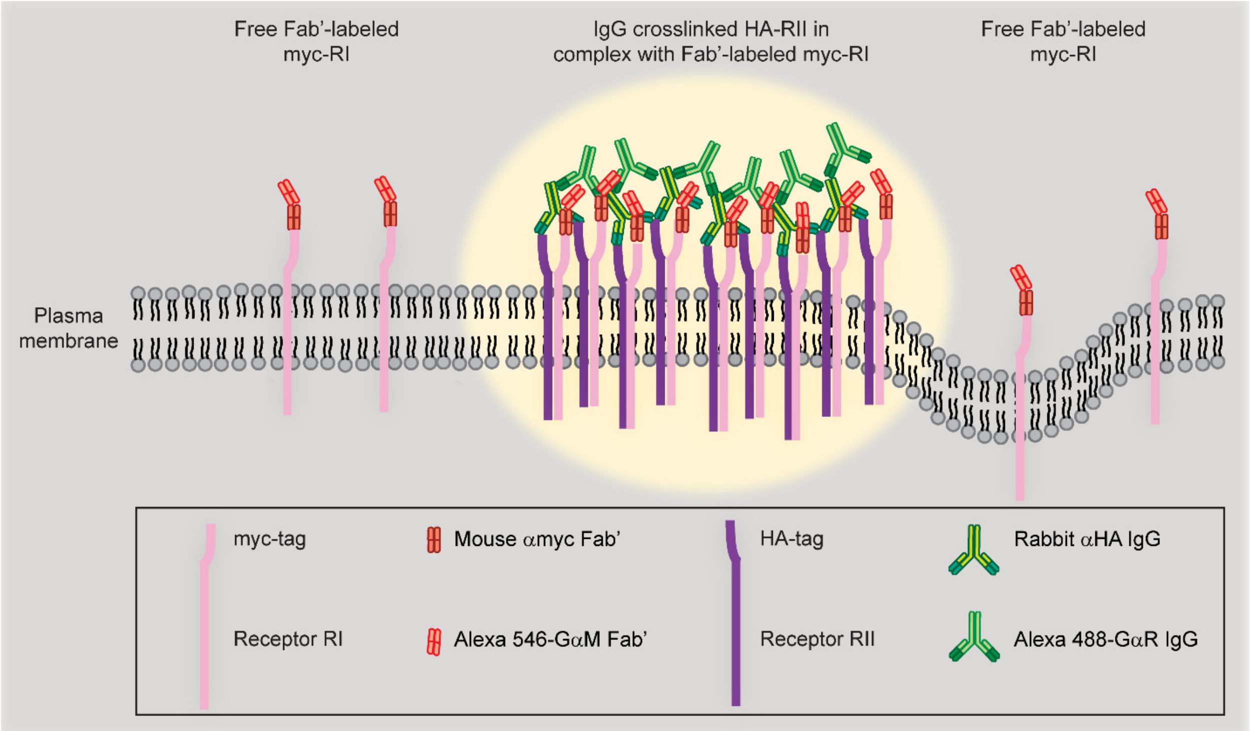

Supplementary Fig. 12 Schematic diagram of patch-FRAP experiments. Two receptors carrying different extracellular epitope tags (here, myc-RI and HA-RII) are coexpressed in the same cells. One receptor (*e.g.*, HA-RII) is labeled at 4 °C (to minimize internalization) by a double layer of rabbit αHA IgG followed by Alexa 488-GαR IgG. This induces crosslinking of HA-RII, inducing its lateral immobilization. Concomitantly, myc-RI is labeled by monovalent Fab' fragments of IgG from another species (murine αmyc Fab'), followed by Alexa 546-GαM Fab' (red fluorescence). Then, the lateral diffusion of the red Fab'-labeled myc-RI is measured by FRAP. In the case that RI and RII form complexes at the cell surface, a fraction of the Fab'-labeled myc-RI would bind to the IgG-immobilized HA-RII. If the complexes are stable on the FRAP timescale, the bound Fab'-myc-RI would be immobilized as well, leading to a reduction in their R_f value. On the other hand, if the RI/RII complexes are transient on the FRAP timescale, R_f of the myc-RI would not be affected; rather, its lateral diffusion coefficient (D) will be reduced, since each myc-RI molecule would be bound to the immobilized HA-RII for part of the time, but will be free to diffuse during the periods in which it dissociates from the immobilized complexes[92].

Supplementary Fig. 13 Original uncropped western blots for all signaling experiments

Prior probing the blots by the indicated antibodies, the membranes were cut in order to allow parallel processing of the different antigens with the respective antibodies

Fig. 5a

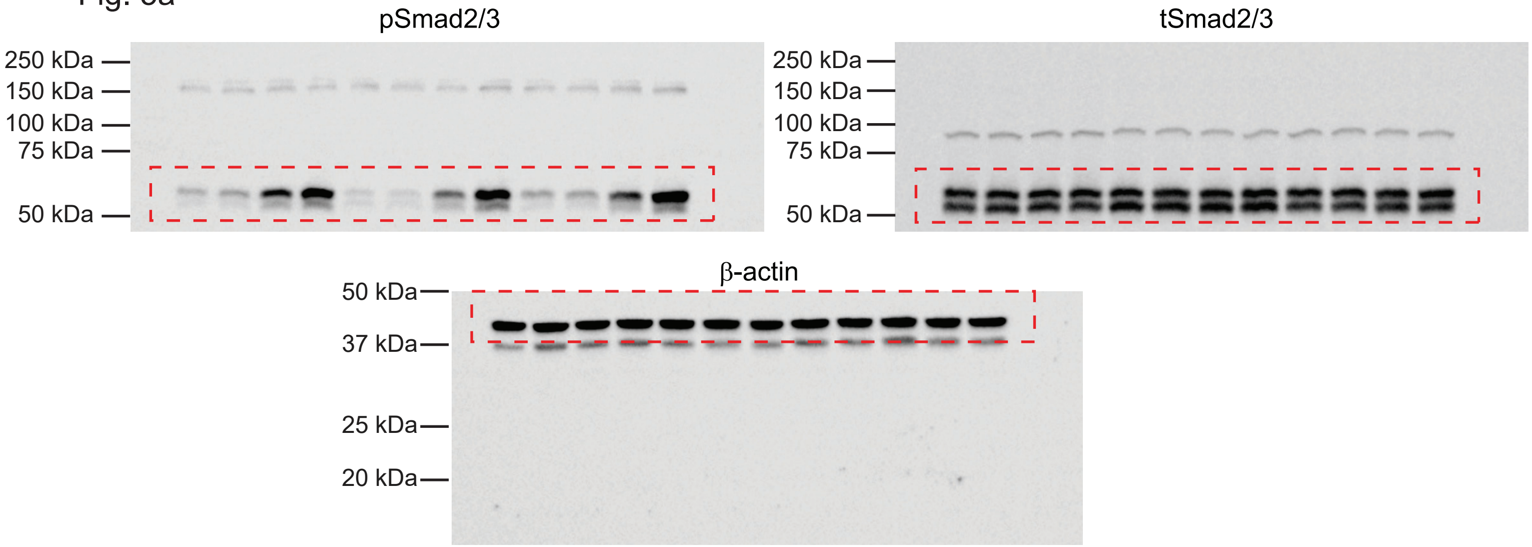

Fig. 6a

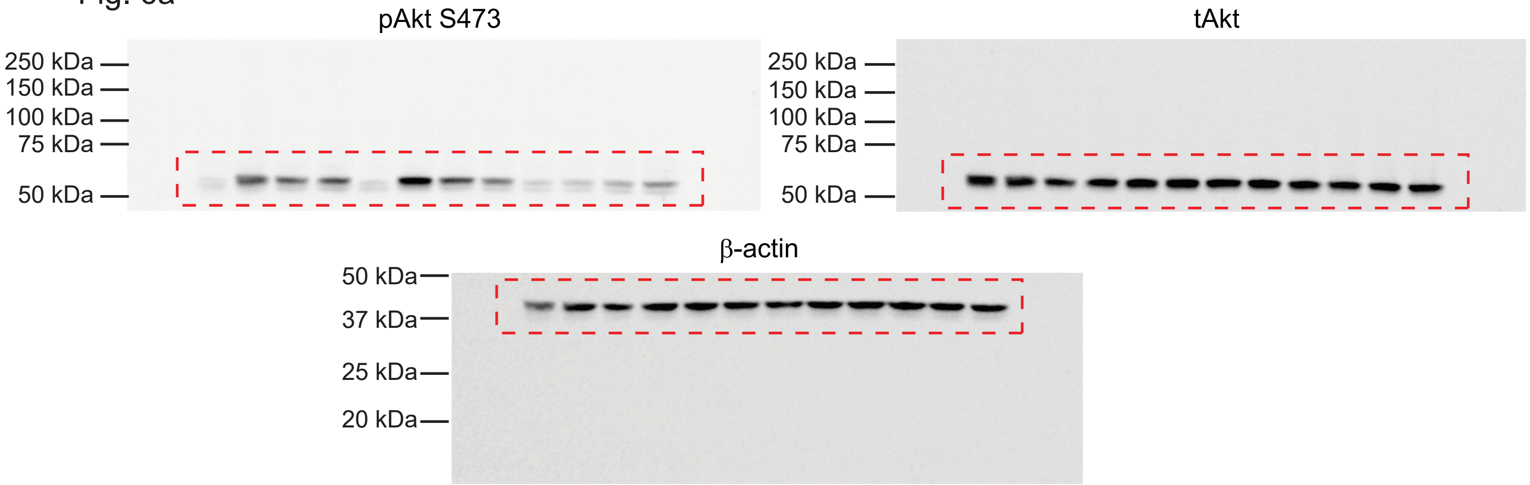

Fig. 6c

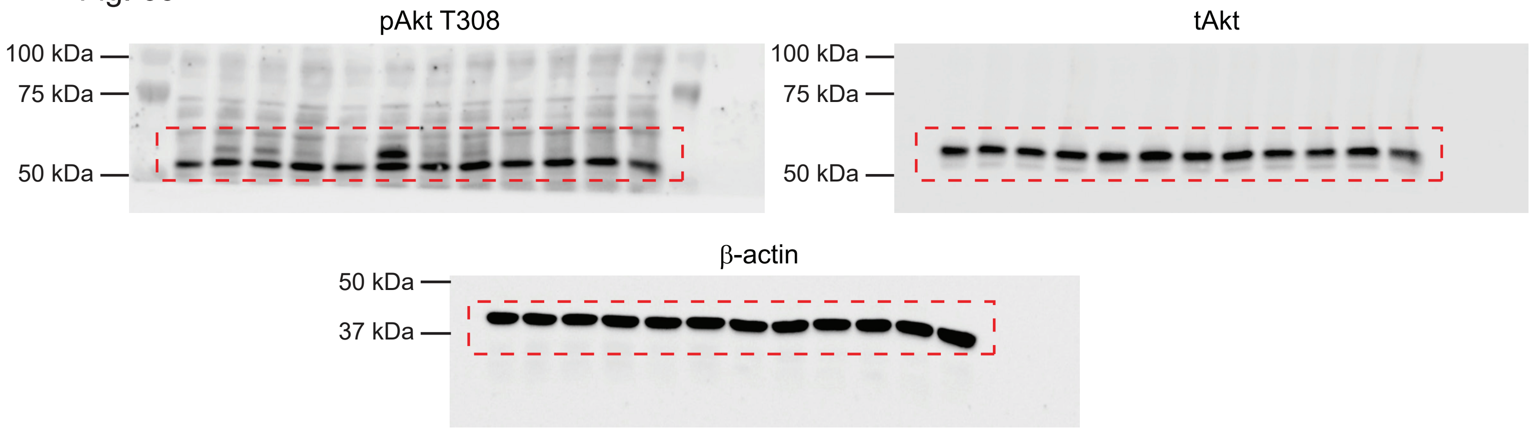

Fig. 7a

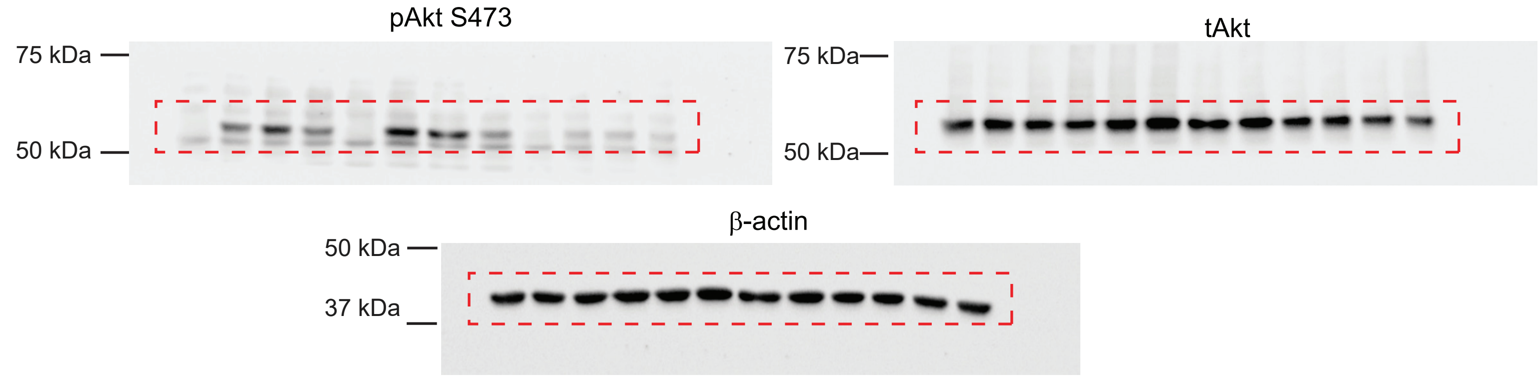

Fig. 7c

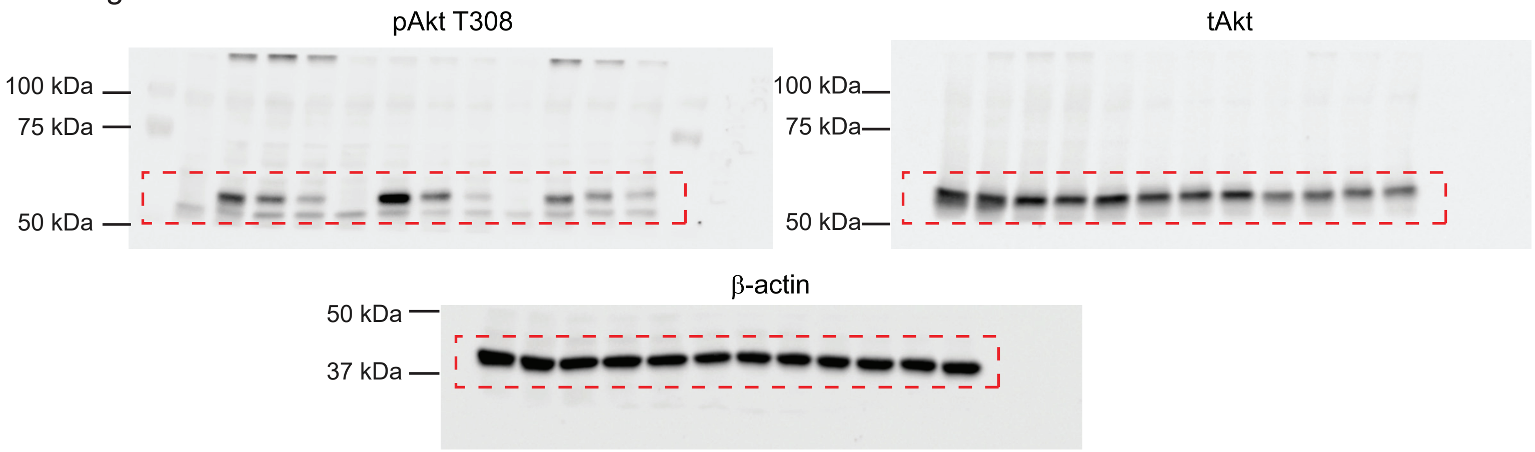

Fig. 8 b

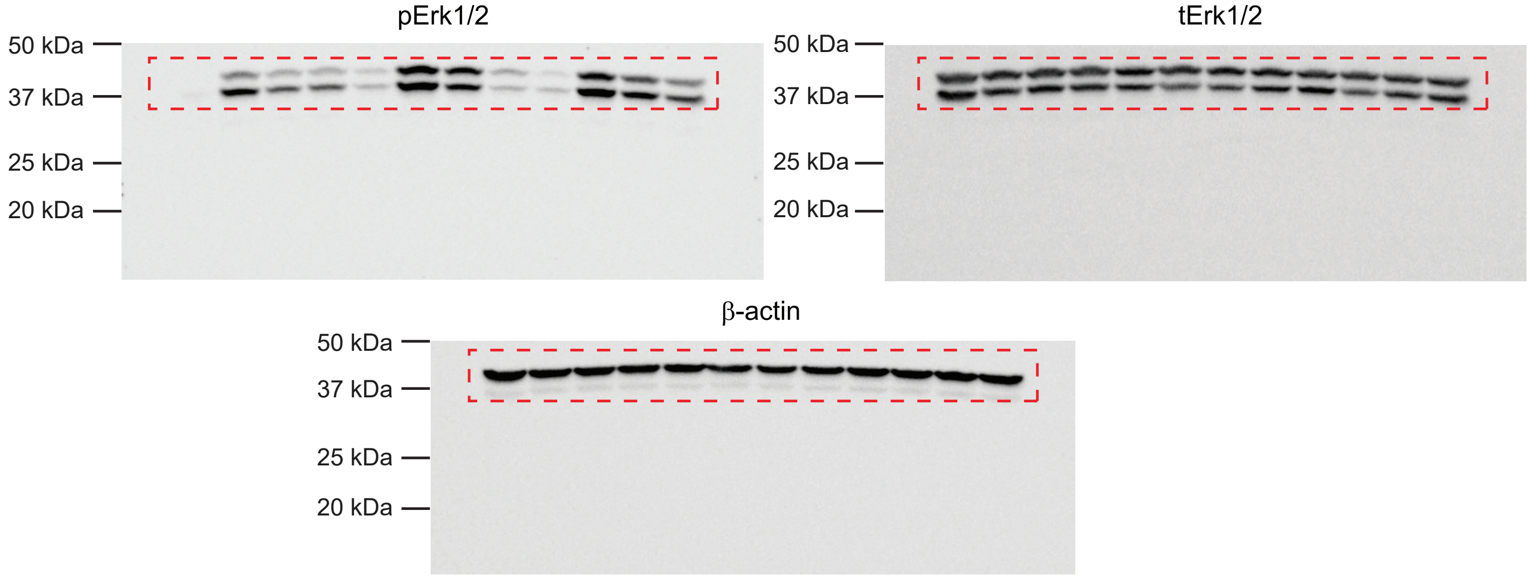

Supplementary Fig. 2a

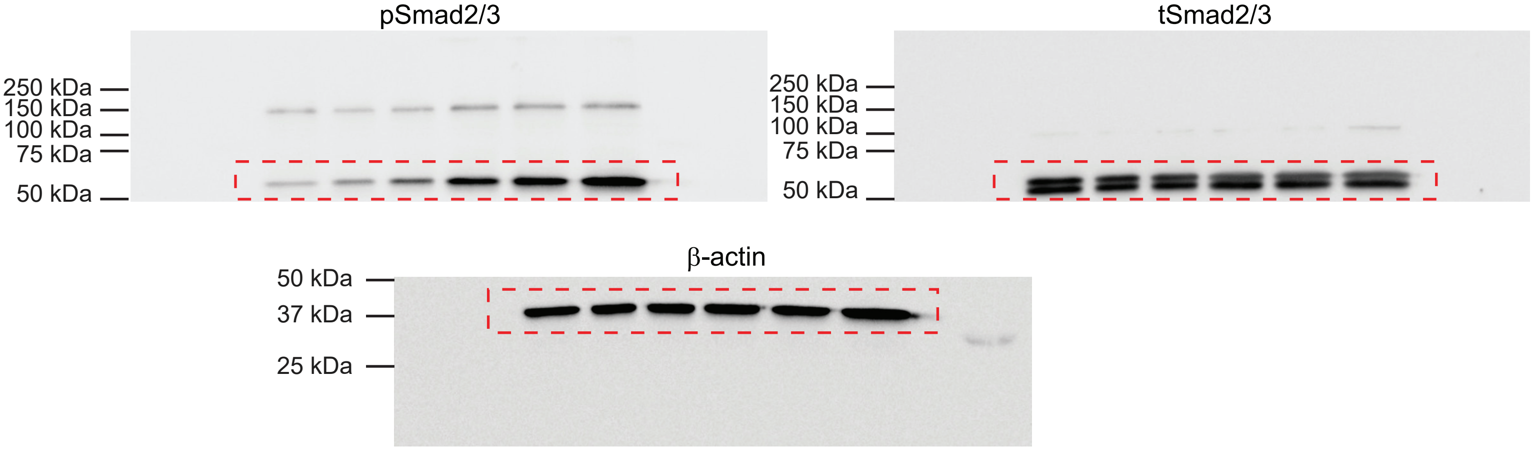

Supplementary Fig. 10a

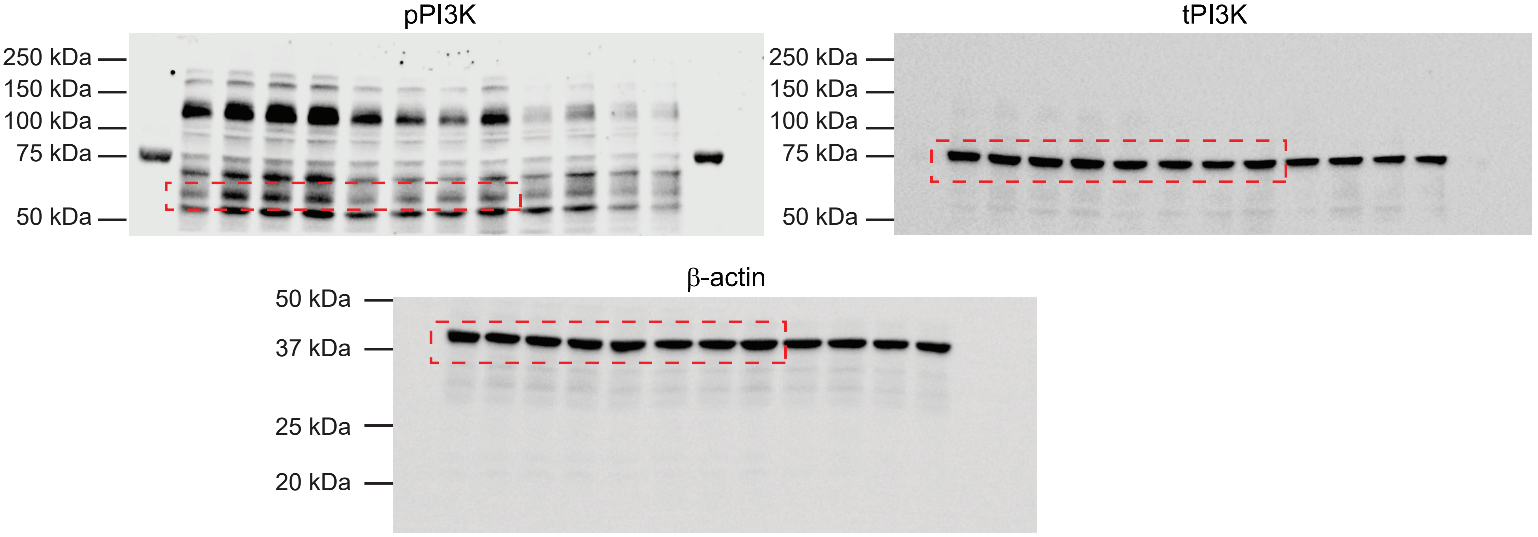

Supplementary Fig. 11a

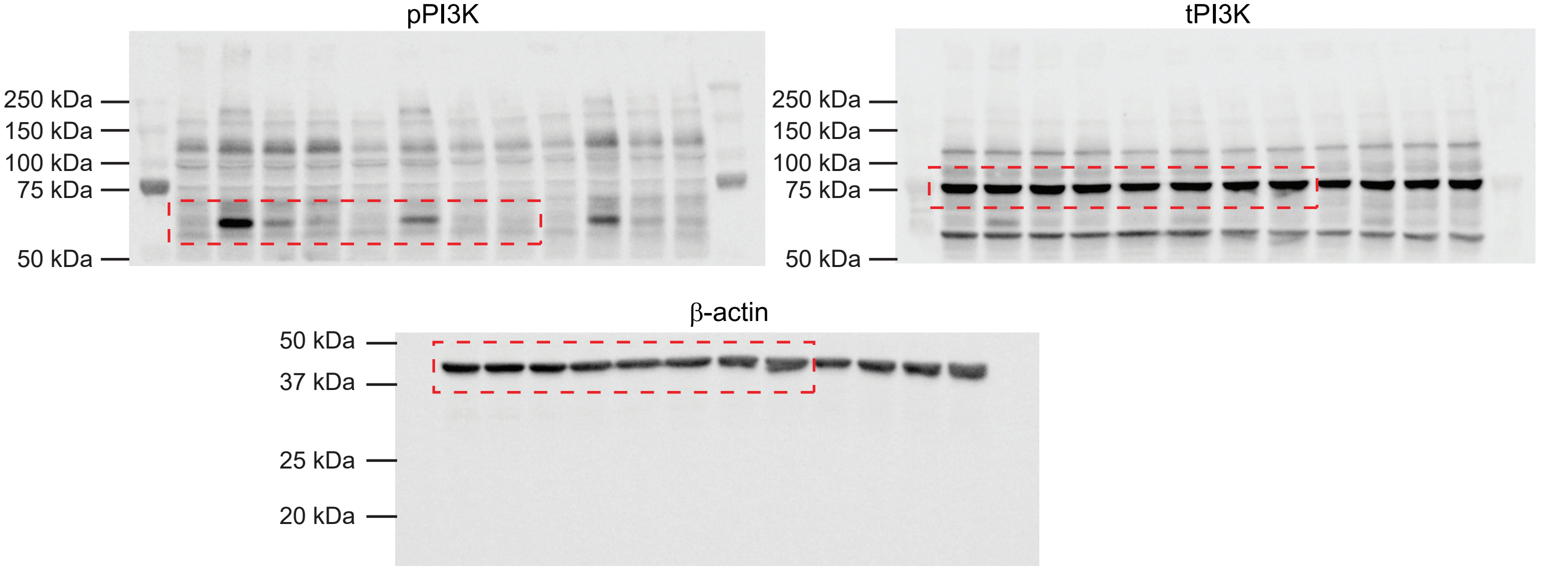

Supplement: Supplementary file 1 — Supplementary information [file 42003_2023_5654_MOESM1_ESM.pdf]
