# Supplementary material for: Cholesterol modulates type I/II TGF-β receptor complexes and alters the balance between Smad and Akt signaling in hepatocytes
Source: Commun Biol. 2024 Jan 2;7:8. doi: 10.1038/s42003-023-05654-9 (PMC10761706; doi:10.1038/s42003-023-05654-9)
Supplement: Supplementary file 2 — Description of Additional Supplementary Files [file 42003_2023_5654_MOESM2_ESM.pdf]

Description of Additional Supplementary Files

File name: Supplementary Data
Description: The source data behind all graphs in the manuscript.
