# Supplementary material for: Cholesterol modulates type I/II TGF-β receptor complexes and alters the balance between Smad and Akt signaling in hepatocytes
Source: Commun Biol. 2024 Jan 2;7:8. doi: 10.1038/s42003-023-05654-9 (PMC10761706; doi:10.1038/s42003-023-05654-9)
Supplement: Supplementary file 4 — Reporting Summary [file 42003_2023_5654_MOESM4_ESM.pdf]

Corresponding author(s): Yoav I. Henis
Last updated by author(s): Nov 24, 2023

Reporting Summary

Nature Portfolio wishes to improve the reproducibility of the work that we publish. This form provides structure for consistency and transparency in reporting. For further information on Nature Portfolio policies, see our Editorial Policies and the Editorial Policy Checklist.

Statistics

For all statistical analyses, confirm that the following items are present in the figure legend, table legend, main text, or Methods section.

n/a	Confirmed	
☐	☒	The exact sample size (*n*) for each experimental group/condition, given as a discrete number and unit of measurement
☐	☒	A statement on whether measurements were taken from distinct samples or whether the same sample was measured repeatedly
☐	☒	The statistical test(s) used AND whether they are one- or two-sided<br>*Only common tests should be described solely by name; describe more complex techniques in the Methods section.*
☒	☐	A description of all covariates tested
☐	☒	A description of any assumptions or corrections, such as tests of normality and adjustment for multiple comparisons
☒	☐	A full description of the statistical parameters including central tendency (e.g. means) or other basic estimates (e.g. regression coefficient) AND variation (e.g. standard deviation) or associated estimates of uncertainty (e.g. confidence intervals)
☐	☒	For null hypothesis testing, the test statistic (e.g. *F*, *t*, *r*) with confidence intervals, effect sizes, degrees of freedom and *P* value noted<br>*Give P values as exact values whenever suitable.*
☒	☐	For Bayesian analysis, information on the choice of priors and Markov chain Monte Carlo settings
☒	☐	For hierarchical and complex designs, identification of the appropriate level for tests and full reporting of outcomes
☒	☐	Estimates of effect sizes (e.g. Cohen's *d*, Pearson's *r*), indicating how they were calculated

Our web collection on statistics for biologists contains articles on many of the points above.

Software and code

Policy information about availability of computer code

Data collection	No software was used
Data analysis	Prism 9 was used for statistical analysis

For manuscripts utilizing custom algorithms or software that are central to the research but not yet described in published literature, software must be made available to editors and reviewers. We strongly encourage code deposition in a community repository (e.g. GitHub). See the Nature Portfolio guidelines for submitting code & software for further information.

Data

Policy information about availability of data

All manuscripts must include a data availability statement. This statement should provide the following information, where applicable:

- Accession codes, unique identifiers, or web links for publicly available datasets
- A description of any restrictions on data availability
- For clinical datasets or third party data, please ensure that the statement adheres to our policy

All data generated or analyzed during this study are included in this published article and its supplementary information files. The Supplementary Information file contains all supplementary figures and the original uncropped Western blots. The source data behind all graphs in the manuscript are in the Supplementary Data file

Research involving human participants, their data, or biological material

Policy information about studies with human participants or human data. See also policy information about sex, gender (identity/presentation), and sexual orientation and race, ethnicity and racism.

Reporting on sex and gender	NA
Reporting on race, ethnicity, or other socially relevant groupings	NA
Population characteristics	NA
Recruitment	NA
Ethics oversight	NA

Note that full information on the approval of the study protocol must also be provided in the manuscript.

Field-specific reporting

Please select the one below that is the best fit for your research. If you are not sure, read the appropriate sections before making your selection.

☒ Life sciences ☐ Behavioural & social sciences ☐ Ecological, evolutionary & environmental sciences

For a reference copy of the document with all sections, see nature.com/documents/nr-reporting-summary-flat.pdf

Life sciences study design

All studies must disclose on these points even when the disclosure is negative.

Sample size	Sample sizes are indicated in each figure legend. The sample size was determined according to the experiment type. For fluorescence recovery after photobleaching studies, a minimum number of 26 cells (with each measurement conducted on a different cell) was measured for each sample, since in our experience with this method (extending over 30 years), about 15 measurements are required to obtain accurate reproducible results. For immunoblots , we used n equal to or greater than 4.
Data exclusions	No data was excluded from the analysis.
Replication	Yes, attempts at replication were successful.
Randomization	Allocation of cells for the experiments was random, as they were split and plated at random while preparing all samples, including control samples.
Blinding	No blinding was used, as all samples were prepared simultaneously for the experiment from the same cell stock.

Reporting for specific materials, systems and methods

We require information from authors about some types of materials, experimental systems and methods used in many studies. Here, indicate whether each material, system or method listed is relevant to your study. If you are not sure if a list item applies to your research, read the appropriate section before selecting a response.

Materials & experimental systems

n/a	Involved in the study	
☐	☒	Antibodies
☐	☒	Eukaryotic cell lines
☒	☐	Palaeontology and archaeology
☒	☐	Animals and other organisms
☒	☐	Clinical data
☒	☐	Dual use research of concern
☒	☐	Plants

Methods

n/a	Involved in the study	
☒	☐	ChIP-seq
☒	☐	Flow cytometry
☒	☐	MRI-based neuroimaging

Antibodies

Antibodies used	12CA5 murine monoclonal IgG anti-HA tag, Roche Diagnostics cat. #11-66-606-001

Antibodies used

9E10 murine monoclonal IgG anti-myc tag, BioLegend cat. #626802
HA.11 rabbit IgG anti-HA tag, BioLegend cat. #923502
Anti-pSmad2/3 rabbit IgG, Cell Signaling Technology cat. #8828
Anti-total (t) Smad2/3 murine IgG, Santa Cruz Biotechnology cat. #sc-133098
Anti-pAkt (phospho-Ser473) rabbit antibody, Cell Signaling Technology cat. #9271
Anti-pAkt (phospho-Thr308) rabbit antibody, Cell Signaling Technology cat. #9275
Anti-tAkt rabbit antibody, Cell Signaling Technology cat. #9272
Anti-pPI3K (phospho-PI3K p85 (Tyr458)/p55 (Tyr199) rabbit antibody, Cell Signaling Technology cat. #4228
Anti-tPI3K (total PI3K (p85)) rabbit antibody, Upstate Biotechnology cat. #06497
Anti-pErk1/2 (diphosphorylated Erk1/2) murine monoclonal antibody, Sigma-Aldrich, cat. #M8159
Anti-tErk1/2 rabbit antibody, Cell Signaling Technology cat. #4695S
Anti-beta actin mouse antibody, MP Biomedicals cat. #08691001
Normal goat gamma-globulin, Jackson ImmunoResearch Laboratories cat. #005-000-002
Alexa 488-goat IgG anti-rabbit IgG, Invitrogen-Molecular Probes cat. #R37116
Alexa 546-goat F(ab')2 anti-mouse IgG, Invitrogen-Molecular Probes cat. #A-11018
Alexa 488-goat F(ab')2 anti-rabbit F(ab')2, Invitrogen-Molecular Probes cat. #A-11070
Peroxidase-goat anti-mouse antibody, Jackson ImmunoResearch Laboratories cat. #115-035-062
Peroxidase-goat anti-rabbit antibody, Jackson ImmunoResearch Laboratories cat. #111-035-144

Validation

All validations are on the web sites of the manufacturers and no new unvalidated antibodies were used.

Eukaryotic cell lines

Policy information about cell lines and Sex and Gender in Research

Cell line source(s)

AML12 murine hepatocyte cell line, ATCC cat. #CRL-2254

Authentication

STR profiling and interspecies contamination test for AML12 cells was performed by the cell line authentication service from IDEXX (Kornwestheim, Germany) using the CellCheckTM Mouse system that includes 19 species-specific STR markers

Mycoplasma contamination

Cells were routinely tested for mycoplasma contamination by RT-PCR every 2 months and found negative.

Commonly misidentified lines
(See ICLAC register)

No commonly misidentified cell lines were used.

Plants

Seed stocks

NA

Novel plant genotypes

NA

Authentication

NA
